# Determining hotspots of gaseous criteria air pollutants in Delhi airshed and its association with stubble burning

**DOI:** 10.1038/s41598-023-51140-x

**Published:** 2024-01-10

**Authors:** Nirwan Nirwan, Asfa Siddiqui, Hareef baba shaeb Kannemadugu, Prakash Chauhan, R. P. Singh

**Affiliations:** 1 Urban and Regional Studies Department, Indian Institute of Remote Sensing, Indian Space Research Organisation, 4-Kalidas Road, Dehradun, Uttarakhand 248001 India; 2 National Remote Sensing Centre, Indian Space Research Organisation, Hyderabad, Telangana 500037 India

**Keywords:** Climate sciences, Environmental sciences, Natural hazards

## Abstract

Transboundary pollutant transport is considered as one of the primary factors causing the seasonal air quality deterioration in Delhi, India’s capital. The highest standard deviations exceeding days in winter for NO_2_ (7.14–9.63%) and SO_2_ (4.04–7.42%) in 2019–2022 underscore the role of meteorological conditions in Delhi's pollution. In contrast, the post-monsoon season shows the highest pollutant exceedance days (4.52–8.00%) for CO due to stubble burning (SB) in Punjab (68,902 fires/year). Despite the government’s assertions of decreasing SB events (14.68%), the city’s CO exceedance days persistently rose by 6.36%. CAMS data is used for assessing contribution hotspots through back-trajectory analysis at multiple heights. An overlap hotspot of 111 sq. km area is identified in the Southeast parts of Punjab that have a higher contribution to the CO levels in Delhi during the post-monsoon season of 2019. Similarly, hotspots are also observed for SO_2_ over industrial areas of Punjab during the post-monsoon and pre-monsoon seasons. The same seasons show similar contributing patterns for NO_2_ highlighting the influence of consistent emission patterns and meteorological conditions. The clear delineation of hotspots using the receptor model at multiple heights coupled with source apportionment studies will assist decision-makers in addressing the pollution sources outside Delhi.

## Introduction

Air pollution is the world's leading cause of deaths among human beings, which is responsible for 7 million fatalities every year. This number is more than the combined premature deaths from heart diseases, stroke, diabetes, road injury and war^1,2^. The increased intensity of anthropogenic activities has led to increased air pollution exposure and vulnerability of the human population^3^. Exposure to air pollution in India is identified as the third major risk factor for human deaths, leading to 12.5% of total deaths in India^4–6^. Furthermore, severe air pollutant conditions lead to fog, dense haze, and smog in the densely populated Indo-Gangetic Plains^7^. Delhi is the most populated city in the region, where the exposure to high levels of air pollution has decreased it’s inhabitants’ life expectancy by 6.4 years^1^. Delhi’s air pollution levels are aggravated due to multifaceted interactions between atmospheric conditions and the various sources of emissions in Delhi’s airshed extending up to Pakistan^8^. *Stubble burning* (SB) or *crop residue burning* (CRB) is regarded as the primary contributor of emissions based on variable factors, including quantity of biomass generated, emission factors, and the impact of different seasons in Northern India^9–14^. The transboundary pollution scenario, specifically in Delhi, due to stubble burning, is a matter of great concern. It is estimated that Delhi receives about 50–75% of its pollutants from SB in post-monsoon season^15^. These emissions are a result of 30.5 million tons of combined SB in Punjab and Haryana^16^. These SB cases have increased over the years (250 per year from 2003 to 2017)^17^ as a result of policies by the Central government, Government of Punjab and Haryana oriented towards conserving ground water by delaying paddy sowing seasons (Prevention and Control of Pollution Act 1981; The Punjab Preservation of Subsoil Act 2009; The Haryana Preservation of Subsoil Act 2009; National Policy for Management of Crop Residue 2014; National Green Tribunal 2015). These delays compel farmers to burn stubble on their lands and prepare their fields for the next crop season^18^.

The number of SB events have decreased by around 11.01% and 41.69% in Punjab from 2018 as compared to 2017 and 2016, respectively^19^. The reduced number of SB cases may not directly lead to reduced pollutant levels in Delhi. Studies have shown that SB is not the only parameter determining the pollutants’ loading in a region such as Delhi^20^. Furthermore, wind speed at the source, favorable wind direction, conducive temperature, and lower wind speed at the receptor play a vital role as well^20^. The complex and heterogeneous distribution of the extent of influence of various factors promoting SB and pollutant transport makes it important to identify the relatively higher contributing areas outside of Delhi. Determining such areas can play a vital role in developing region-specific policies.

SB may emit a significant quantity of air pollutants such as CO_2_, N_2_O, CH_4_, CO, NO_x_, SO_2_, and particulate matter (PM_2.5_ and PM_10_) like elemental carbon^11,21^. Sahai et al.^22^ established that burning 63 Mt of stubble biomass releases about 91 Mt of CO_2_, 3.4 Mt of CO, 0.6 Mt of CH_4_, 0.1 of NO_x_, and about 1.2 Mt of particulate matter into the atmosphere. The abovementioned pollutants are the primary emissions of many other activities prevalent in North West India. Various researchers have extensively studied the association between SB and particulate matter; however, very little impetus has been made on the study of gaseous criteria air pollutants and their sources in the Northwest India^15,18,20,23–25^. While, CO, NO_2_ and SO_2_ are emitted due to SB, Carbon Monoxide (CO) and Nitrogen Dioxide (NO_2_) are also released due to incomplete fossil fuel combustion from motorized transport. Burning of sulphur-rich fossil fuels such as coal significantly contributes to sulphur dioxide (SO_2_). The long-term increase in stubble burning and industrialization in the region has translated to increasing gaseous pollution trends in Delhi from 2013 to 2019^26^. The stringent policies oriented towards shifting to fuel-efficient mobility engines have led to a decrease in nitrogen dioxide (NO_2_) levels in the city from 2012 to 2019^26,27^. However, the pollutant levels show seasonal peaks in November and December every year^26^. The annual and seasonal variations of these pollutants (CO, NO_2_, and SO_2_) in conjunction with the high vulnerability of Delhi’s population, emphasizes the need to study the pollutant transport in detail.

Multiple studies have previously adopted WRF-Chem model to study the transport of pollutants (PM_2.5_, PM_10_ and NO_2_) from source based approach, limiting the studies by administrative boundaries to India^28–30,31^. Researchers have used receptor-based air parcel trajectories developed through HYSPLIT model to spatially identify the pollutant sources, especially for PM_2.5_ and black carbon in the northwest India^18,24,25^. While general studies on transboundary pollutant transport use ground data to determine the pollutant carrying trajectories (received at 100–500 m AGL) using Concentration Weighted Trajectory (CWT) analysis^24^, this study uses modelled satellite data to assess backward pollutant trajectories.

Therefore, this study attempts to analyse the weekly spatio-temporal variations in gaseous criteria air pollutants (CO, NO_2_, and SO_2_) during 2018–2022 using satellite-based measurements from Sentinel-5P TROPOMI datasets over northwest India. Since gaseous air pollutants and their association with stubble burning are sparingly explored in literature, the study further investigates the role of stubble burning and meteorological factors (wind direction, air temperature, and planetary boundary height) on the seasonal contribution hotspots for the gaseous pollutants using weighted Concentrated Weighted Trajectory (WCWT) analysis. The study uses Copernicus Atmosphere Monitoring Service (CAMS) global reanalysis product to assess pollutant trajectories from 100 to 1500 m AGL, corresponding to the area’s mixing height^32^. These results are further compared to ground-level data (100 m AGL) for the same period to justify the adoption of a modeled dataset. The identified hotspots are interrogated for their potential overlap with high emission anthropogenic activities such as active fires. These spatial overlaps assist in spatially segregating the activities with similar characteristics based on their pollutant contribution towards Delhi due to varying meteorological conditions.

### Site description

Delhi is situated between the rich rain-washed Indo-Gangetic Plain (IGP) in the East and the semi-arid tracts of Rajasthan to the South-west. It is one of the most densely populated and polluted urban environments^33^ over the globe owing to a high population density (11,297 persons per sq. km)^34–36^. The administrative area of the National Capital Territory (NCT) Delhi is located between Haryana and Uttarakhand, as depicted in Supplementary Fig. S1. The city is divided into three segments: flood plain, ridge and upper Gangetic plain^37,38^. It is characterized by a monsoon influenced humid subtropical climate (Cwa) bordering a hot semi-arid climate (BSh) as per the Köppen classification system^39^. The city experiences four seasons, viz. pre-monsoon (PrM) (March through May), monsoon (June through September), post-monsoon (PoM) (October and November) and winter (December through February). The air temperature in the region varies from 4–10 ℃ in winters to 42–48 ℃ in summers^40,41^. Gentle south-east flowing winds are experienced during the monsoon season, and the northwest winds blow across the region during the rest of the year with an intensity of 2–5 ms^−1^^7^. The orography of the region facilitates restricted wind circulation and low air pollution dispersion, converting Delhi into a hotspot of urban and regional pollution. Significant sources of gaseous air pollution in the region include emissions from thermal power plants, transportation, small-scale industries (such as brick kilns), domestic cooking, and seasonal agricultural biomass burning^7,42^.

## Material and methods

**Figure 1:**
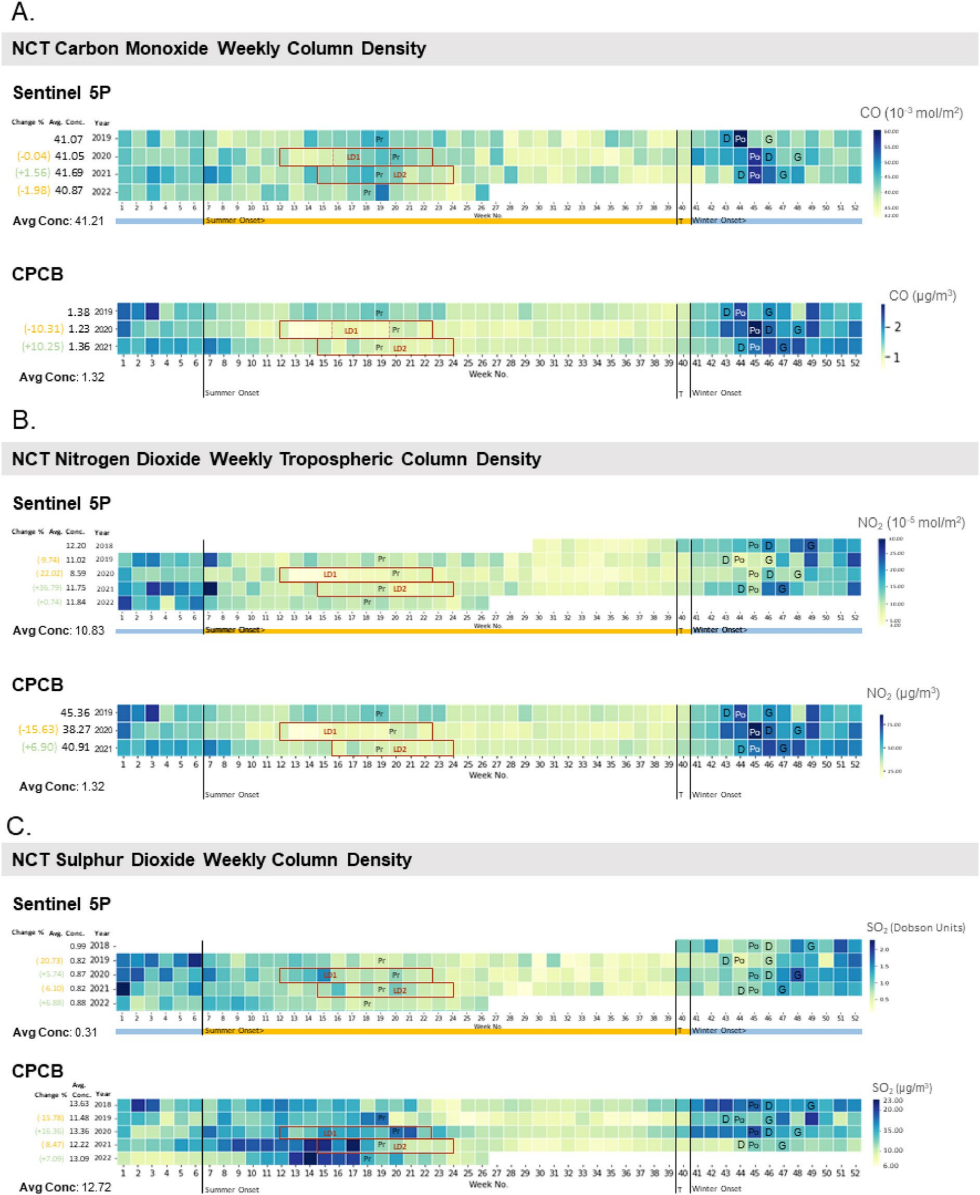
Trends of criteria air pollutants; (**A**) Carbon Monoxide (CO), (**B**) Nitrogen Dioxide (NO_2_) and (**C**) Sulphur Dioxide (SO_2_); observed from satellite based (Sentinel-5P TROPOMI) and ground-based monitoring stations’ (CPCB) data in NCT of Delhi. *Note*: The nomenclature corresponds to: LD1- March 22 to May 31, 2020; LD2: April 05 to June 15, 2021; PrM: Pre-monsoon stubble burning peak in Punjab; PoM: Post-monsoon stubble burning peak in Punjab; D: Diwali festival; G: Gurupurab festival; T: Procurement of Kharif crops from farmers (Oct 1).

*Gaseous air pollutants’ trends* The weekly concentration of identified criteria air pollutants viz. Carbon Monoxide (CO), Nitrogen Dioxide (NO_2_) and Sulphur Dioxide (SO_2_) is assessed using datasets from the European Space Agency’s (ESA) Sentinel-5 Precursor (Sentinel-5P) TROPOspheric Monitoring Instrument (TROPOMI) from 2019 to 2022 (Supplementary Table S1). The offline stream (OFFL) level 3 data is extracted using cloud based system called Google Earth Engine (GEE) API platform^43^. The Copernicus satellite mission is capable of measuring several trace gases at a spatial resolution of 3.5 × 7 sq. km (for NO_2_ and SO_2_) and 7 × 7 sq. km (for CO) with the help of three spectrometers covering the ultraviolet-near infrared region at 270–500 nm and 675–775 nm, and one spectrometer covering the shortwave infrared region (SWIR)^44^. The differential optical absorption spectroscopy (DOAS) method is used for extracting the tropospheric NO_2_ and total column SO_2_, whereas, a modified SWIR CO retrieval (SICOR) approach is applied for retrieval of CO^41,44,45^. Weekly concentration composites are developed into heat maps to be assessed simultaneously with anthropogenic events. Developing weekly composites further removes data gaps from pixel drop in area of interest. To validate the results from satellite-based measurements, daily pollutant concentration levels are collected from 39 central pollution control board (CPCB) (http://app.cpcbccr.com/ccr/#/caaqm-dashboard-all/caaqm-landing/caaqm-comparison-data) ground-based monitoring stations (Supplementary Table S2) are plotted similarly for the study area. The plots boxplots are created using R studio and combined with the line charts using MS PowerPoint (Supplementary Figs. S4, S5, S6, S7, S8, S9, S10, S11S12). The heatmap for different years are generated using Python (Fig. 1). The variations in pollutant levels are compared to potentially contributing events such as stubble burning, seasonal variations in planetary boundary layer (PBL) and temperature, pollution causing festivals such as Diwali and Gurupurab, and COVID lockdowns.

*Fire data* The stubble burning events are studied using Fire Information for Resource Management System (FIRMS) that provides the thermal anomaly and location information at 1 × 1 sq. km. using the MODIS Collection 6 NRT Hotspot / Active Fire Detections available under the tag “MCD14DL”. The SB points are defined by incorporating pixels with confidence level greater than 30% as laid out by the product manual and multiple studies^18,46^. It is important to note that many studies use an 80% confidence level along with a 320 K temperature threshold to attain better detection accuracy and segregate forest fires from SB^47,48^. This higher accuracy leads to a lower detection rate, as the user guidelines mention. We observed that adopting the later approach in IGP leads to non-conclusive trends and relatively lower fire count as compared to statistics observed in government records and other studies^18,46,49,50^.

The weekly mean is calculated for Punjab and Haryana to be compared to the pollutant levels in NCT. In addition to fire count, seasonal and annual fire density maps are prepared during the study period (2019 which showed minimal bias due to natural causes and pandemic) categorized as Low Fire Prone region (LFP) (50–150 fires), Moderate Fire Prone region (MFP) (150–300 fires), High Fire Prone region (300–500 fires) and Intense Fire Prone region (IFP) (fires > 500). The seasonal analysis of pollutants and fire density/count is assessed for winter (December through February), pre-monsoon (March through May), Monsoon (June through September) and post-monsoon (October and November) and the annual analysis is from December 2018 to November 2019, hereafter referred as 2019.

*Meteorological parameters* The meteorological data is crucial for determining the dependance of pollutant levels. Hence, it is sourced from two primary repositories: the Central Pollution Control Board (CPCB) of India and the ERA 5 (European Centre for Medium-Range Weather Forecasts Reanalysis) dataset.

The CPCB, as a statutory body, diligently collects and maintains a repository of meteorological parameters within the Indian subcontinent. This dataset encompasses critical variables such as temperature, net solar radiation, wind speed and direction, etc. The CPCB data is downloaded from the official government portal (http://app.cpcbccr.com/ccr/#/caaqm-dashboard-all/caaqm-landing) for 39 stations in Delhi. The data is restructured, and the average values of all observations are considered for analysis.

Complementing this ground level dataset, the study also integrates information from ERA 5, which represents the fifth generation of global reanalysis data provided by the ECMWF. ERA 5 comprehensively depicts various meteorological parameters at a 0.25° spatial resolution on a global scale, including but not limited to temperature, mixing height, wind speed and direction and net solar radiation. The data is available in a NetCDF format, which is converted into series data for comparable analysis with the pollutant concentration data.

*Concentration weighted trajectory (CWT) analysis* An integrated approach is adopted to identify the spatial areas contributing to different pollutants in the study area. The CWT analysis can cluster air mass back trajectories (developed using HYSPLIT model) into trajectories with similar pollutant carrying characteristics. The TrajStat extension in MeteoInfo software (http://meteothink.org/) is used to develop back trajectories for different vertical profiles of the atmosphere^51^, combining meteorological measurements retrieved from GDAS (https://www.ready.noaa.gov/gdas1.php). The trajectories are classified according to contribution level by incorporating the corresponding pollutant levels. The trajectory calculation involves multiple parameters such as the start date of trajectory computation, the point of origin, the height of origin point (AGL), the hour of beginning the trajectory, the number of hours to trace back, meteorological variables and end date. For this study, the geometrical center of NCT is assessed at 100 m, 500 m, 1000 m and 1500 m AGL for different seasons. The model computes the trajectories for 168 h, 72 h and 38 h for CO, NO_2_, and SO_2_ respectively, as per the lifetimes of pollutants defined by IPCC^52^.

The hourly pollution concentration data is added to the trajectory files to compute CWT using ground- and satellite-based measurements. The pollutant concentration at different heights is acquired from Copernicus Atmosphere Monitoring Service (CAMS) reanalysis product. The data product integrates model data with worldwide observations into a globally wide-ranging and consistent dataset utilizing a physics and chemistry-based atmosphere model. A grid of spatial resolution 0.25° is used to compute the results from trajectories to the spatial grid with CWT values using the following equation:

$${C}_{ij}= \frac{1}{{\sum }_{l=1}^{M}{\tau }_{ijl}}{\sum }_{l=1}^{M}{C}_{l}{\tau }_{ijl}$$

where ${C}_{l}$ is the observed concentration at the advent of trajectory $l$, τ represents the time spent by trajectory $l$ in $ij$th cell. Here,$i$ and $j$ are used as index to identify the cell while $l$ is an index for trajectories with $M$ being the total number of trajectories. The higher values of ${C}_{ij}$ relate to higher potential of the trajectories over the cell to carry pollutants to the receptor site.

Weight concentration weighted trajectory (WCWT) values are used to reduce the impact of minimal values. The ground-based pollution concentration data is computed and compared with 100 m arrival height CWT results with satellite-derived results. Composite maps at proposed heights for different seasons are developed to visualize and assess the spatial variability of the contributing areas for different pollutants. To maintain continuity in data, the seasons are defined from December 2018 to November 2019. The seasonal assessment is carried out in pre-monsoon season (March–May i.e., week 10–22), monsoon season (June–September i.e., week 23–39), post-monsoon season (October–November i.e., week 40–48) and winter season (December 2018, week 49 to February 2019, week 9). The resultant maps are then compared to the fire density maps developed for annual and seasonal periods using FIRMS data. All maps are plotted using licensed software ArcMap 10.5. The detailed methodology flowchart is represented in Supplementary Fig. S2.

*Statistical tests*: Correlation is a statistical measure that quantifies the linear relationship between two variables. It is denoted by the coefficient 'r'. The coefficient ranges from − 1 to + 1, signifying the strength and direction of the association. A positive correlation (r > 0) implies a simultaneous increase in both variables, while a negative correlation (r < 0) denotes an inverse relationship.

The Sen Slope is a statistical linear regression method used when dealing with outliers or data irregularities. It is ideal for time series data as it calculates the median of slopes between pairs of data points, offering a robust measure of the trend. The Sen Slope improves accuracy in trend analysis by minimizing the impact of extreme values.

## Results

### Trend of pollutant concentration

The pollutant trends are assessed from 2018 to 2022 (based on data availability) for CO, NO_2_ and SO_2_. These pollutant trends are compared to meteorological parameters and anthropogenic events on annual and seasonal scale.

*Carbon monoxide (CO)* There was a marginal decline of 0.04% observed from 2019 to 2020 and a slight increase of 1.56% from 2020 to 2021. These findings are presented in Fig. 1, illustrating the average and standard deviation for different years and seasons. The varying trends in pollutant concentration over the assessed years is attributed to variation in anthropogenic activities such as COVID-19 induced lockdowns^53^. These lockdowns were imposed in India from 22nd March to 31st May 2020 (LD1) and 5th April to 15th June 2021 (LD2). Additionally, these lockdowns led to shift in the sowing seasons for Kharif crops^54^. It is essential to note that the weekly observation data does not reflect the impact of other anthropogenic activities such as festivals. As Mukherjee et al., 2018 concluded, the influence of events like Diwali on pollutant levels is more pronounced in daily measurements due to their susceptibility to changes in meteorological conditions^55^. The impact of festive events on average daily pollutant levels is still evident, as shown in Supplementary Fig. S3.

**Figure 2:**
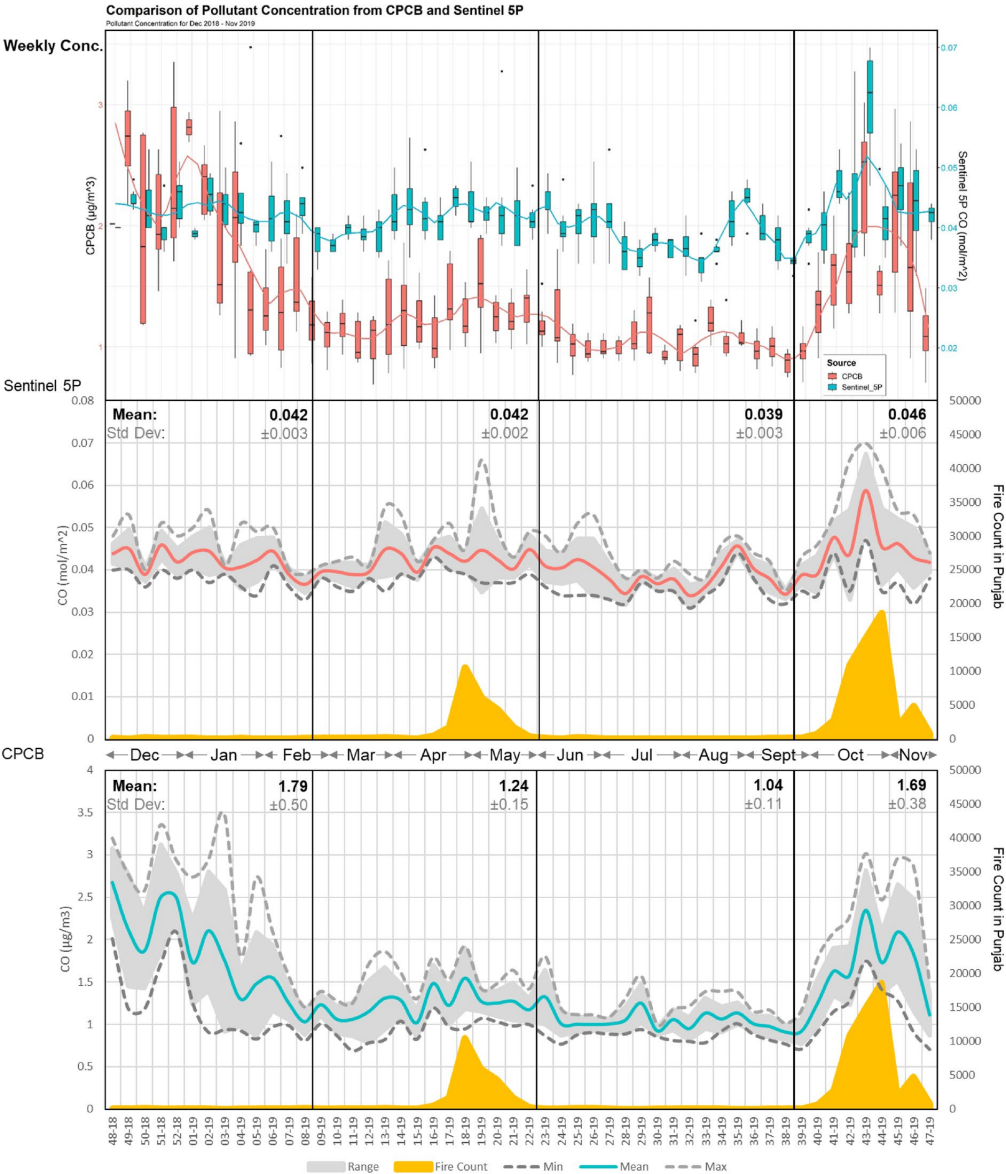
Weekly Trends of Sentinel-5P/CPCB CO in Delhi NCT and MODIS FIMRS derived active fire count in Punjab (December 2018–November 2019).

The reduced levels of CO during monsoon are attributed to several factors. Brick kilns do not operate during monsoon season, and SB is limited to PrM and PoM harvest seasons. The increase in pollutant levels is directly linked to active fires in Punjab in PrM and PoM seasons. This led to surge in the region’s weekly average during peak of SB (week 44 in 2019 and week 45 in 2020 and 2021). Over the past five years (2018–2022), FIRMS-derived fires show an average of 19,430 PrM fires/year and 68,902 PoM fires/year observed in Punjab and 5996 PrM fires/year and 8953 PoM fires/year in Haryana. Notably, the year 2021 recorded the highest average CO concentration over the four years. It is important to note that 2021 is also affected by COVID 19 lockdown (especially LD2), which decreased the number of days exceeding the mean in the PrM and monsoon seasons. Exceedance days are defined as the percentage of days in a time period that observed higher pollutant concentration than the average or first standard deviation (± 1σ). The winter season of 2021 shows the highest exceedance days (6.21%) among all years and seasons, leading to higher annual pollutant concentration (Supplementary Table S3). This increase can be attributed to more fires (83,115) observed in Punjab during Post-Monsoon (PoM) season in 2021. This is higher compared to 2018 (PrM 21,657 and PoM 67,881), 2019 (PrM 24,873 and PoM 56,038), 2020 (PrM 13,500 and PoM 79,563) and 2022 (PrM 23,787 and PoM 57,916). While the PrM season of 2021 observed relatively lower fires (13,335), the impact of PoM SB on the pollutant levels in Delhi remains substantial. This higher impact may be attributed to favorable meteorological conditions^15,20,24,25^. The rise of pollutant levels in Delhi coincides with the peaks of stubble burning in Punjab, as observed in Fig. 2.

The pollutant concentrations assessed using Central Pollution Control Board (CPCB) and Sentinel-5P TROPOMI for CO show a moderately positive correlation (r = 0.62). The variance between the two datasets is explained by CPCB collecting the pollutant levels close to the ground. At the same time, Sentinel 5P provides total column density of the pollutant in the atmosphere. The correlation coefficient increases to 0.81 and 0.67 in the PoM and winter seasons. The average planetary boundary layer (PBL) is also low during PoM (418.00 ± 80.82 m) and winter (332.04 ± 80.98 m) seasons. This is lower than the average annual PBL of 536.47 ± 233.74 m (Supplementary Fig. S4). This further conforms with the lower average seasonal temperatures of 23.95 °C and 14.09 °C in the PoM and winter season, respectively. Temperature and PBL are highly correlated (r = 0.87), and show a moderately strong inverse relationship with the CPCB and satellite data (r = − 0.36 and − 0.53, respectively) (Supplementary Figs. S5 and S6). The comparatively strong seasonal correlation of anthropogenic activities compared to meteorological variables establishes SB as the influential factor affecting the pollutant levels in Delhi.

To analyze the variation in pollutant concentration during different seasons and years, the days exceeding the mean values is presented in Supplementary Table S3. The results are expressed as a percentage of days when the values exceeded the annual mean and the sum of mean and standard deviation. In the years 2019, 2020, 2021, and 2022, data was available for 310 days (84.93%), 325 days (89.04%), 322 days (88.22%) and 322 days (88.22%), respectively, due to instances of pixel drop in the area of interest. The year 2021 shows the highest percent of exceedance days due to the higher number of active fires observed in 2021, as mentioned earlier. The winter and PoM seasons demonstrated a higher percent of exceedance days for annual mean due to lower weekly mixing heights (217.10 m for winter and 476.91 m for PoM respectively) and temperature (14.33 °C for winter and 16.47 °C for PoM respectively). The PoM season shows the highest percentage of exceedance days above the mean and standard deviation, associating the season with extreme peak pollutant values (primarily due to stubble burning). PrM season also shows higher pollutant exceedance levels from the annual mean due to the PrM stubble burning.

*Nitrogen dioxide (NO*_*2*_*)* The annual average levels of NO_2_ are notably influenced by anthropogenic activities such as mobility, which tend to have relatively consistent emissions throughout the year. A sudden decrease in pollutant levels is observed during LD1 in 2020 as compared to other years (Fig. 1). This reduction can be attributed to restrictions over anthropogenic activities, primarily associated to vehicular movement in the city during lockdown^41^. The NO_2_ concentration observation data from Sentinel 5P indicates a moderate negative correlation (r = − 0.65) with the near-surface air-temperature data collected from ERA 5 for 2019, as shown in Supplementary S12. NO_2_ concentration is closely associated with vehicular emissions, which persist throughout the year^26^.

**Figure 3:**
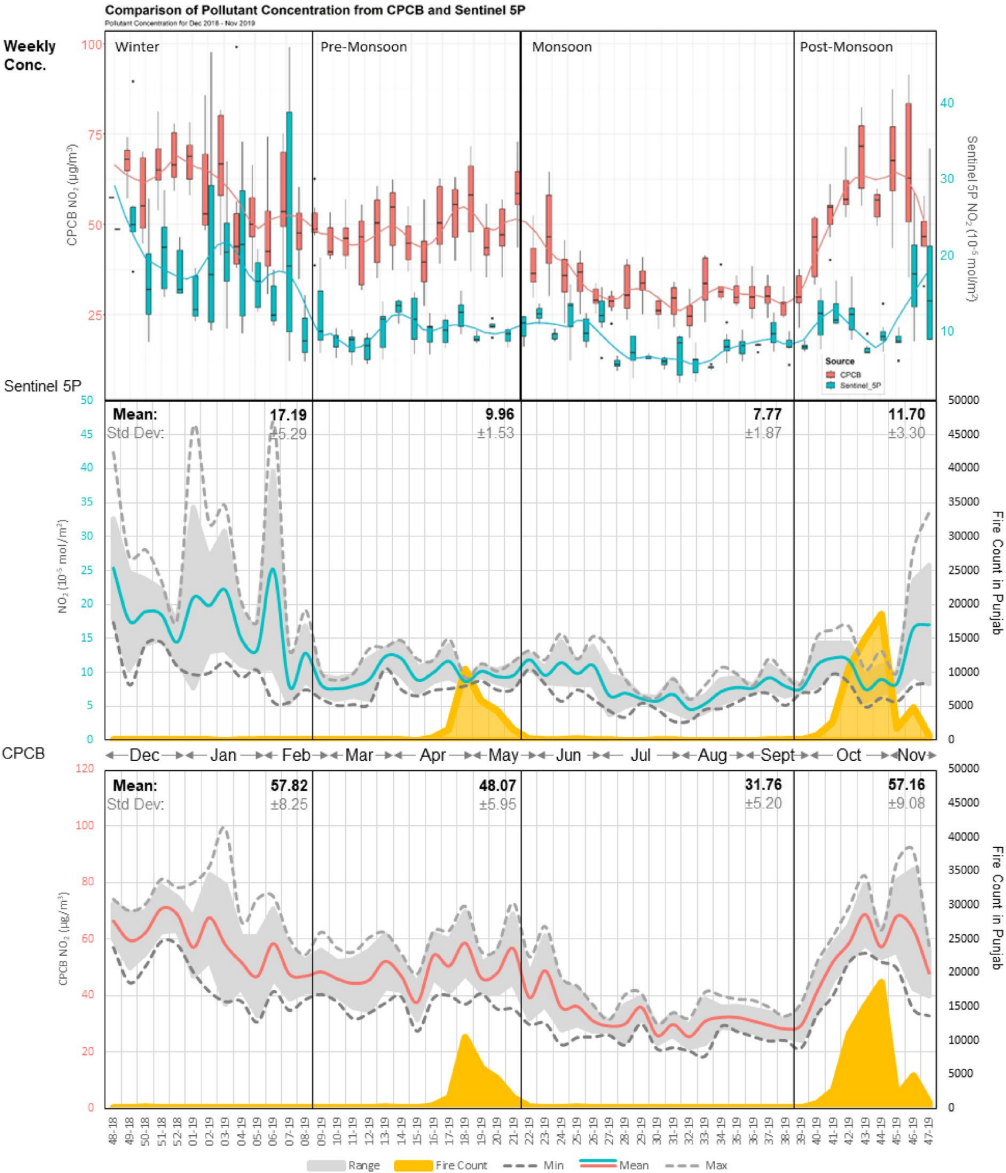
Weekly Trends of Sentinel-5P/CPCB NO_2_ in Delhi NCT and MODIS FIMRS derived active fire count in Punjab (December 2018–November 2019).

As mentioned earlier, the meteorological conditions play a crucial role in the variability of NO_2_ levels. This leads to an average pollutant concentration of 16.06 ± 4.90 × 10^–5^ mol/m^2^ and 11.64 ± 3.06 × 10^–5^ mol/m^2^ during winter and PoM seasons, respectively, from 2018 to 2022. In contrast, the PrM and monsoon seasons exhibit relatively lower levels of 8.65 ± 1.62 × 10^–5^ mol/m^2^ and 7.83 ± 1.80 × 10^–5^ mol/m^2^ for the same study period, respectively. The CPCB NO_2_ levels show a moderate correlation of 0.47 and 0.52 with active fire counts in Punjab during the PrM and PoM season, respectively, as represented in Fig. 3. TROPOMI derived NO_2_ shows moderate to high correlation with ERA5 (r = − 0.65) and CPCB (r = − 0.57) monitored air temperature, justifying higher NO_2_ values during PoM and winter season (Supplementary Fig. S12). The correlation between other meteorological parameters and NO_2_ is seasonal. The CPCB monitored net solar radiation has a positive correlation of 0.56 and 0.79 in PrM and monsoon season, with average values of 168.25 ± 34.64 W/m^2^ and 143.40 ± 33.67 W/m^2^, respectively. However, the correlation inverses during PoM and winter seasons with the average values of 97.68 ± 27.10 W/m^2^ and 93.27 ± 23.22 W/m^2^_,_ leading to a correlation of − 0.57 and − 0.41 respectively (Supplementary Fig. S9). Similar trends are also observed with mixing height data collected from ERA 5, showing a moderately positive correlation of 0.60 and 0.68 for PrM and monsoon seasons. At the same time, there is a negative correlation of -0.66 during the winter season. The PoM season concentration does not show any strong correlation with mixing height due to the shorter duration of the assessment season during this period (Supplementary Fig. S8).

The highest number of exceedance days was observed in 2020 (38.92%) among the years under study, despite the presence of LD1 in PrM season (Supplementary Table S4). This increase in exceedance days can be attributed to the restrictions placed on anthropogenic activities in 2020, leading to lower annual mean and standard deviation values. Consequently, exceedance days are more prevalent in winter and PoM seasons, where the influence of LD1 was relatively low. However, the annual mean had been reduced to 8.59 × 10^–5^ mol/m^2^. The presence of LD1 and LD2 in PrM season of 2020 and 2021 reduced its exceedance days to 3.80% and 3.43% respectively. In contrast, higher exceedance of 6.80% in 2019 and 8.46% in 2022 is observed. The strong association of NO_2_ to meteorological conditions results in higher exceedance days for winter and PoM seasons, specifically for the sum of mean and standard deviation.

*Sulphur dioxide (SO*_*2*_*)* The average annual SO_2_ levels exhibit a degree of variability due to their unique source dependency and low lifetime in the atmosphere. The pollutant levels are quantified in Dobson units (DU) that describe the pollutant’s thickness in hundredths of a millimeter if compressed to constant pressure and temperature^56^. The sensor and CPCB show a moderately positive correlation of 0.51 for the year 2019, which increases to 0.67 during monsoon season when the pollutant levels are at their lowest (0.51 ± 0.15 DU). On the contrary, the winter and PoM season shows the highest level of pollutant concentration and variability of 1.25 ± 0.35 DU and 0.93 ± 0.27 DU, respectively. The PrM season shows a relatively lower pollutant concentration of 0.79 ± 0.19 DU.

**Figure 4:**
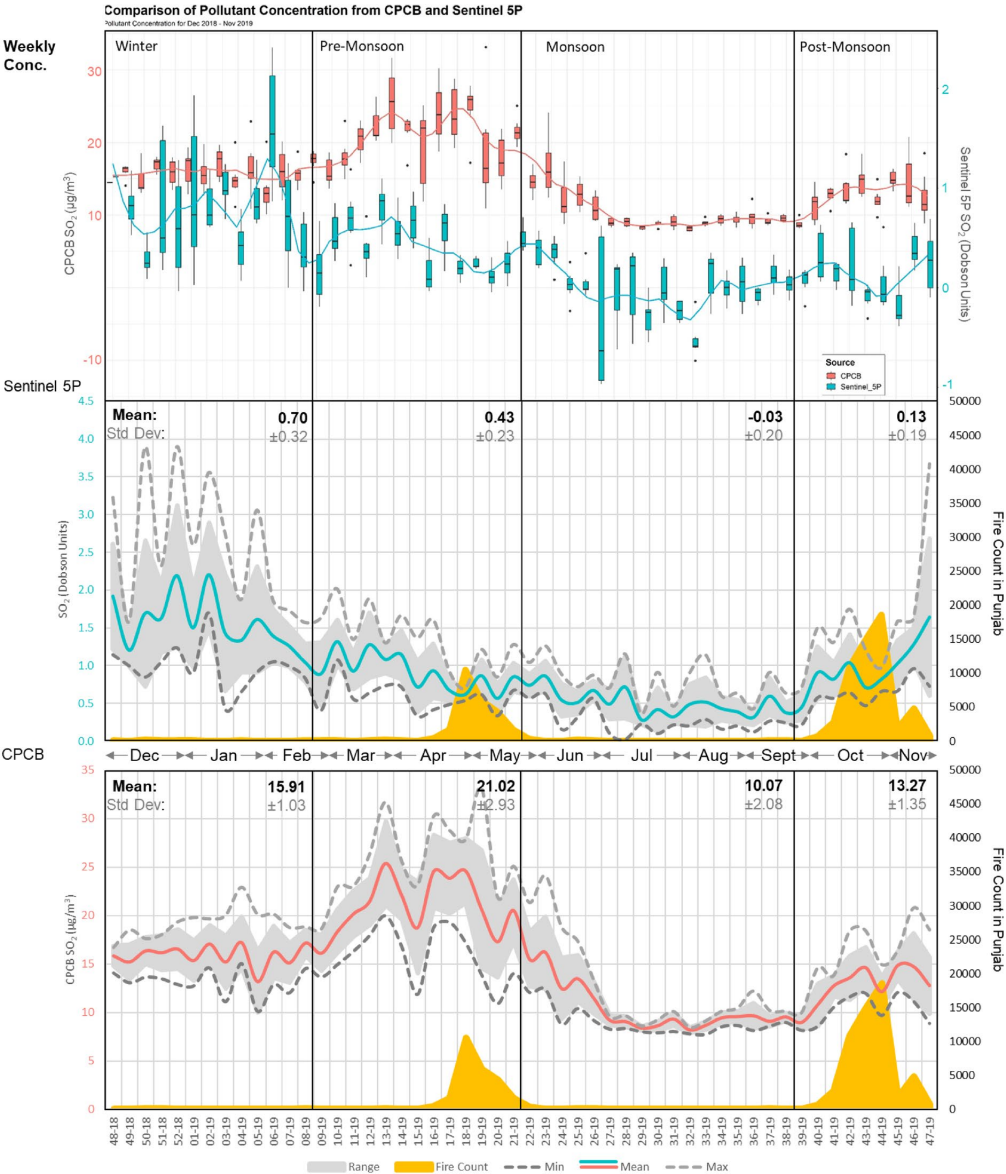
Weekly Trends of Sentinel-5P/CPCB SO_2_ in Delhi NCT and MODIS FIMRS derived active fire count in Punjab (December 2018–November 2019).

The SO_2_ pollutant levels do not exhibit any discernable strong association with stubble burning trends (r < 0.40), as evident in Fig. 4. The SO_2_ concentration strong correlates with meteorological parameters, especially during monsoon season. The pollutant concentration shows a high correlation of 0.88 with air temperature during monsoon season with an average value of 31.30 (± 2.87 °C). This temperature is notably higher than the values observed during winter (14.09 ± 1.64 °C), PrM (28.46 ± 5.50 °C) and PoM (23.96 ± 2.64 °C) seasons (Supplementary Fig. S12). Additionally, the pollutant displays a moderately positive correlation of 0.65 with the net solar radiation during monsoon season (Supplementary Fig. S10) due to scavenging of SO_2_ from precipitation. However, it is expected that less net radiation will result in lower temperatures, resulting in low PBL leading to increase in pollutants. In the monsoon season, precipitation scavenging reduces the surface concentration.

A higher percentage of exceedance days is consistently observed during the winter season. In contrast, the monsoon season shows the least exceedance for mean and mean plus standard deviation, as illustrated in Supplementary Table S5. The decrease in SO_2_ levels can be associated to the non-functional coal-based brick kilns in the surrounding areas during the monsoon season.

The pollutant trends for all three trace gasses strongly correlate to seasonal anthropogenic activities outside of Delhi, including but not limited to SB in Punjab. The strong correlation between mixing height and ground-level trace gas concentration further raises the need to study the pollutant transport at multiple receptor heights. The seasonal nature of pollutants in Delhi also highlights the need to assess the sources of pollution in different seasons. The trend analysis also highlights 2019 as the only year with no anomalies in anthropogenic activities and consistent data availability during analysis.

### Concentration weighted trajectory (CWT) analysis

The potential contributing areas to pollution are analyzed based on the weighted concentration-weighted trajectory (WCWT) algorithm. It provides the contribution of different areas to the receptor point, i.e., geometric center of the Delhi NCT administrative boundary.

*Carbon monoxide (CO)* The results for the CPCB data at 100 m AGL and CAMS data at 100 m, 500 m, 1000 m and 1500 m AGL are assessed in comprehensible units, i.e. µg/m^3^ and mg/kg respectively. The continuous results are categorized based on the distribution of values in the resulting contribution images. The new classes are then reclassified as Extreme Contributing Areas (ECA) (> 1.00 mg/kg for CAMS and > 1.25 µg/m^3^ for CPCB), High Contributing Areas (HCA) (0.50–1.00 mg/kg for CAMS and 0.75–1.25 µg/m^3^ for CPCB) and Average Contributing Areas (ACA) (0.05–0.5 mg/kg for CAMS and 0.25–0.75 µg/m^3^ for CPCB). It is important to note that these classifications are developed for relative understanding of contribution levels, as the CAMS and CPCB results cannot be compared directly due to the differences in measurement techniques.

During 2019, an area of 1,637.25 km^2^ was observed under High contribution class for CO. The contributing areas decrease with the increase in trajectory origin height, with HCA of 28,610.25 km^2^, 25,197 km^2^ and 15,484.5 km^2^ for 500 m, 1000 m and 1500 m (Table 1). Similar trends are observed for different seasons, with sources distributing in a circular pattern at 1500 m, in contrast to a directional trend at 100 m (Supplementary Fig. S13). The winter and PoM seasons exhibit a higher ECA of 1914.75 km^2^ and 111 km^2^, respectively, in composite results (Supplementary Figs. S14 and S17). However, no ECAs observed during PrM and monsoon seasons (Supplementary Figs. S15 and S16). The annual WCWT results show ECA lying in the Punjab (Pakistan), Punjab, Haryana, Himachal Pradesh and Uttarakhand. The latter states do not have any known significant sources of pollution, such as stubble burning and brick kilns. The hotspots are visible in the hilly areas due to the higher interaction of air trajectories with the lower Himalayas rather than originating from the source itself.

**Table 1:**
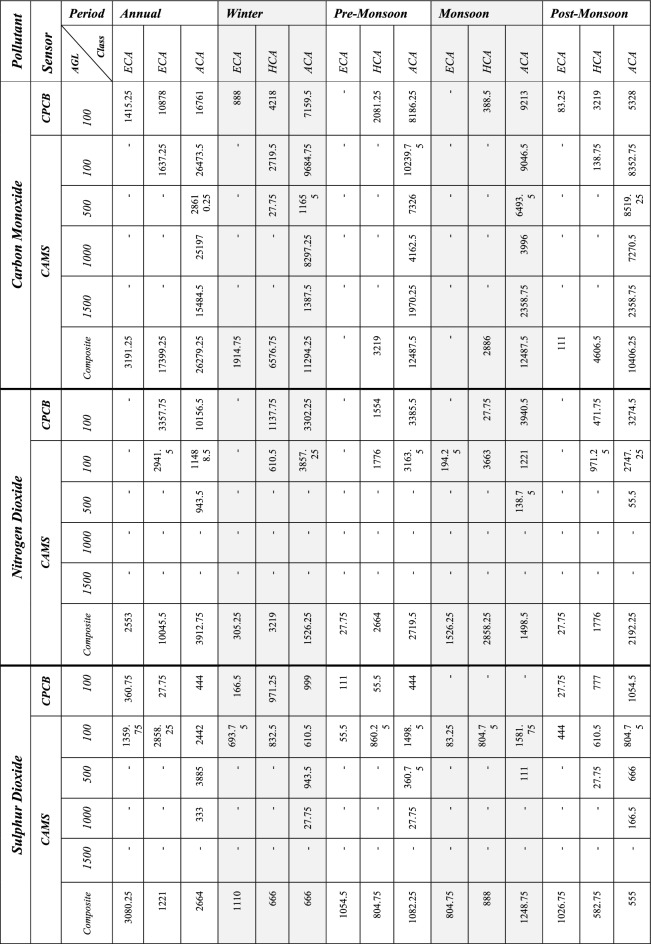
Area (in km sq.) under seasonal contribution classes at varying heights (in m) for CO, NO_2_ and SO_2_.

**Figure 5:**
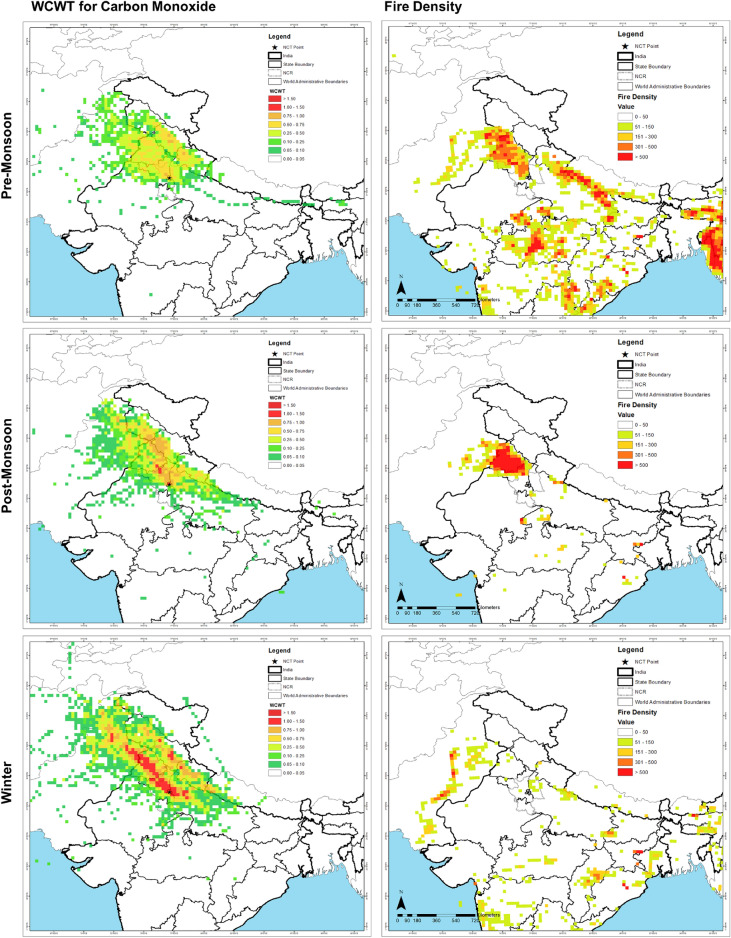
WCWT Results for different seasons for CO and corresponding fire density maps. Maps are generated using opensource software MeteoInfoMap (http://meteothink.org) and ArcMap 10.5.

The overlap between the WCWT results and Fire Density maps (Supplementary Fig. S18) is examined to assess the region’s spatial distribution of contributing SB cases. The PrM season has no ECA but the HCA shows an overlap of 388.5 km^2^ and 693.75 km^2^ with Intense Fire Prone (IFP) and High Fire Prone (HFP) regions, mainly distributed over Punjab and Haryana (Fig. 5). The high contributing area for the PrM season also overlaps with regions of Moderate Fire Prone (MFP) and Low Fire Prone (LFP) area, encompassing 1082.25 km^2^ and 1110 km^2^, respectively. The ECA overlaps 83.25 km^2^ and 138.75 km^2^ with the IFP and HFP in the southeast and central parts of Punjab during PoM season (Fig. 5).

It is important to note that almost the entire Punjab lies in the IFP zone, but only a small part lies in the ECA. Additionally, the high contributing areas show an overlap of 915.75 km^2^, 111 km^2^, 499.5 km^2^ and 222 km^2^ for IFP, HFP, MFP and LFP distributed majorly over the South-West and Central Punjab as well as North Haryana. These results help us directly identify areas that contribute more to Delhi’s pollutant levels as compared to the others. However, during Winter season, the WCWT results show the most extensive area under the ECA (1914.75 km^2^) among the assessed seasons. The ECA of the winter season shows no overlap with the IFP and HFP areas; instead, there are barely any fires observed in Punjab during that period (Fig. 5).

*Nitrogen dioxide (NO*_*2*_*)* The NO_2_ gas is relatively less abundant compared to CO and is studied using different units for CAMS (µg/kg) and CPCB (µg/m^3^) data. The ECA is defined by WCWT results showing values > 6 µg/kg for CAMS and > 50 µg/m^3^ for CPCB data. They have a WCWT resultant value ranging from 3.00 to 6.00 µg/kg for CAMS and 30.00 to 50.00 µg/m^3^ for CPCB The ACA is classified by WCWT values of 1.00–3.00 µg/kg for CAMS and 10.00–30.00 µg/m^3^ for CPCB.

**Figure 6:**
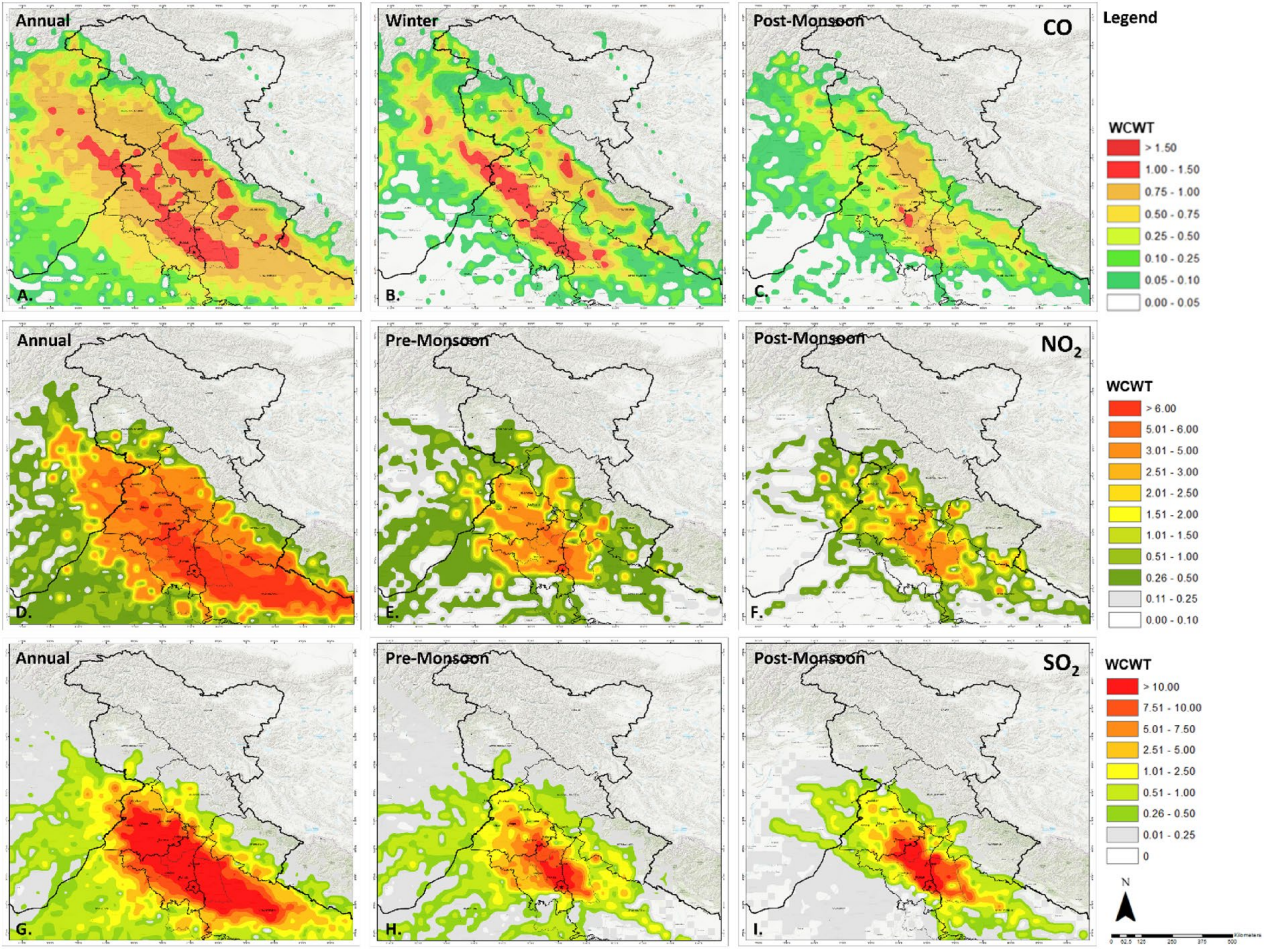
WCWT composite annual using CAMS data (**A**), Winter (**B**) and Post monsoon (**C**) WCWT results for carbon monoxide; composite annual (**D**), pre-monsoon (**E**) and post monsoon (**F**) WCWT results for nitrogen dioxide; composite annual (**G**), pre-monsoon (**J**) and post-monsoon (**I**). Maps are generated using opensource software MeteoInfoMap (http://meteothink.org) and ArcMap 10.5.

Since NO_2_ is a short-lived gas, the observed pollutant contribution areas for 1500m and 1000m AGL are minimal (Supplementary Fig. S19). ECA is primarily observed in composite results distributed along Delhi, Haryana and Uttar Pradesh plains. The area under HCA for CPCB and CAMS at 100 AGL is 3357.75 km^2^ and 2941.5 km^2^ respectively. This area further increases to 10,156.5 km^2^ and 11,488.5 km^2^ for ACA (Supplementary Fig. S19). The composite monsoon season shows the highest area under ECA and HCA of 1526.25 km^2^ and 2858.25 km^2^. This change is attributed to the shift in preceding wind patterns from east to west, leading to the development of larger contributing areas towards the east of NCT (Fig. 6). The meteorological conditions play a crucial role in the transport of pollutants. Consequently, the composite winter season exhibits relatively larger ECA and HCA of 305.25 km^2^ and 3219 km^2^, respectively. While most of the contributing areas are located in and around the Delhi region, an ACA of 1526.25 km^2^ is spread over Punjab and Haryana in the North-West direction (Supplementary Fig. S20). The PrM and PoM seasons show relatively similar contribution area patterns, with an area of 27.75 km^2^ for ECA, 2664 km^2^ (PrM) and 1776 km^2^ (PoM) for HCA, along with 2719.5 km^2^ (PrM) and 2192.25 km^2^ (PoM) for ACA (Table 1) (Supplementary Figs. S21 and S23). A specific belt of contributing areas is observed in PrM and PoM season stretching over Sangrur, Kaithal and Panipat.

Trend analysis for NO_2_ gas shows a strong association of the pollutant with meteorological parameters. The PrM and PoM seasons show similar contribution area patterns in 2019 (Fig. 6). In contrast, winter and monsoon seasons contribute substantially from the north-west and east directions, respectively (Supplementary Figs. S20 and S22).

*Sulphur dioxide (SO*_*2*_*)* The WCWT results for SO_2_ are computed in µg/kg for CAMS data and µg/m^3^ for CPCB data, considering different heights and periods. SO_2_, being a short-lived gas with the highest molecular weight (g/mol) among the assessed gasses, exhibits relatively less association between CAMS model data and localized ground observation data. WCWT resultant values greater than 7.50 µg/kg for CAMS and 7.50 µg/m^3^ for CPCB are classified as ECA. The HCA are defined from 5.00 to 7.50 µg/kg for CAMS and 5.00–7.50 µg/m^3^ for CPCB, while the ACA is defined by value ranges of 2.50–5.00 µg/kg for CAMS and 2.50–5.00 µg/m^3^ for CPCB.

Similar to NO_2_, the SO_2_ gas does not exhibit ECA or HCA for 500 m, 1000 m, and 1500 m AGL due to its short lifespan and molar weight (Supplementary Fig. S24). However, the PoM season is the only season that shows an area of 27.75 km^2^ as an HCA for a height of 500 m AGL (Supplementary Fig. S28). When examining the annual composite WCWT results, an area of 3080.25 km^2^ is identified as ECA, along with 1221.00 km^2^ for HCA. Additionally, the results show an area of 2664.00 km^2^ for composite ACA (Supplementary Fig. S24). The winter season shows the highest contributing area of 1110.00 km^2^ and 666.00 km^2^ for both HCA and ACA (Supplementary Fig. S25). The PrM and PoM seasons show relatively similar contribution extents with an area of 1054 km^2^ and 1026.75 km^2^ for ECA (Table 1) (Supplementary Figs. S26 and S28). The monsoon season exhibits relatively lower contribution areas in the ECA, covering 804.75 km^2^, while HCA spans over 888 km^2^ and ACA encompass 1248 km^2^, which is highest among its class. The results show that the contributing areas in the eastern region are relatively evenly distributed. Notably, almost the entire state of Punjab and Haryana contribute to the SO_2_ levels in Delhi throughout the year (Fig. 6).

The contribution patterns for all three pollutants highlight the role of anthropogenic activities and meteorological parameters during different seasons at varying heights. The contribution areas for CO and SO_2_ highlight the areas with higher contributing anthropogenic activities in Punjab and Haryana. The results for NO_2_ show consistent contribution patterns for PrM and PoM due to consistent emission patterns from primary sources, leading to a more substantial influence of meteorological parameters.

## Discussion

This study deliberates the impact of peak PoM SB in Punjab on the pollutant levels in Delhi, a phenomenon previously corroborated by multiple studies^12,57–59^. This impact is particularly pronounced in the case of CO levels in Delhi. It is noteworthy that a general increase in pollutant levels is observed during the initial weeks of October. This period coincides with the decrease in temperature, marking the onset of winter, and the initiation of government markets for the acquisition of rice grains from farmers (T, Fig. 1) The winter and PoM season exhibit a higher percentage of exceedance days for annual mean pollutant levels due to the influence of lower mixing heights and lower temperature.

The PoM season shows the highest percent of exceedance days above the mean and standard deviation associating the season to extreme peak pollutant values (as a result of SB in the surrounding regions). Similarly, the PrM season shows higher pollutant exceedance levels from the annual mean due to the PrM stubble burning. Since higher exceedance is not observed in mean and standard deviation, it is understood that the pollutant levels do not reach extreme values in the season. The above notion may be questioned by the fact that 2021 exhibits the highest percentage of mean and standard deviation exceedance days in the winter season i.e., 6.21%, while 2020 shows the highest exceedance of 8% during the PoM season. However, PoM SB cases are higher in 2021 than in 2020. It is important to note that Beig et al.^20^ have previously established that the amount of biomass burnt in Punjab is not directly proportional to the pollutant levels observed in Delhi.

A noticeable spike in the CO levels is observed around the PoM SB periods in Punjab. A relatively stronger association is observed between SB and CO levels as compared to meteorological conditions. Similar findings have also been established by multiple research articles elucidating the impact of different meteorological parameters^42,60^. However, it is important to note that pollutant levels remain consistently elevated throughout the winter season. The term “elevated” is used instead of increasing, as the Thein Sen slope from September to November 2019 shows a value of 0.01. This value is not indicative of any overall increasing or decreasing trend once the peak stubble burning values are excluded. These statistics align with previous studies that emphasize the critical roles of anthropogenic activities and meteorological conditions in determining the pollutant levels in Delhi^7,15,18,20,24,25,61^.

The transport of pollutants from the North-Western parts of Delhi is facilitated by low mixing height, low temperatures and supporting wind profile in PoM season as observed in Supplementary Fig. S29. Similar enabling conditions are also observed during the winter season, which has a larger area encompassed by ECA. The winter season exhibits higher exceedance for all years despite the lack of any notable seasonal anthropogenic activities that may contribute to such high levels. The winter season has the largest area categorized as ECA (1914.75 km^2^), which shows no overlap with the IFP and HFP areas. Fewer fires are observed in Punjab during winter (Fig. 5). This suggests an important fact: Though more pollutants may be emitted during PoM season due to stubble burning, more pollutants are transported from surrounding areas during the winter season. This further justifies the consistently elevated pollution levels observed in Delhi during the winter season.

The contributing area patterns identified in this study at 100m have been observed by other studies for different years^7,18,24,25^. The integration of CAMS data in WCWT and multiple height analysis leads to clear delineation of contributing area hotspots. The annual composite ECA cover cities in Punjab (Pakistan) such as Shekhupura, Narowal and Lahore. This belt further extends through Punjab (India), encompassing cities like Amritsar, Moga, Hoshiarpur, Barnala and Patiala along with Kaithal and Jind in Haryana. During the PoM season, the hotspot outside Delhi is primarily located around Sangrur (Punjab) and Kaithal (Haryana) (Fig. 6).

A specific belt of contributing areas is observed in PrM and PoM season that stretches over Sangrur, Kaithal and Panipat. As established through trend assessment, it is evident that the NO_2_ gas is strongly influenced by meteorological conditions, leading to similar pollutant transport patterns among the PrM and PoM seasons (Fig. 6). In contrast, the winter and monsoon seasons show a more substantial contribution from the northwest and east directions, respectively (Supplementary Figs. S20 and S21). A significant part of state of Punjab and Haryana contributes to the pollutant levels of SO_2_ in NCT throughout the year (Fig. 6). However, cities including Jalandhar, Ludhiana, and Moga emerge as HCA hotspots during low temperatures in PoM and winter seasons. These areas have dedicated industrial areas that may be potential sources for gasses like SO_2_. The contributing areas shift from northwest to east during monsoon season due to change in prevalent wind directions, as observed by Sarkar et al.^23^.

## Conclusion

This study identifies the spatial distribution of contributing area for gaseous criteria air pollutants such as stubble burning. The CO levels in NCT show a sudden increase, that coincides with the SB peaks of Punjab in the PoM season. The SB events in the PoM harvest season leads to highest pollutant exceedance for CO (4.52%-8.00%) from 2019 to 2022. The marginal decline (14.68%) in PoM fire counts in Punjab over the past four years and the increase (6.36%) in exceedance days in Delhi highlights the varying role of SB cases inside the state. The utilization of CAMS data in creating trajectories at multiple heights for the WCWT model assists in clearly determining the contributing areas for a pollutant. The PoM season observes an overlap of 111 km^2^ between the IFP and ECA. It is evident from the spatial contribution pattern that despite the entire state observes IFP cases during the PoM harvest season, only a specific hotspot in the southeast parts of Punjab significantly contributes to the pollutant levels of Delhi. It is necessary to note that there is a substantially larger (1914.75 km^2^) strongly contributing area observed in winter season compared to PoM season, despite minimal SB cases in winter season in the northwest region. This indicates that even though more CO is generated during the PoM season due to SB, a larger area contributes to the pollutant transport during the winter season due to favorable meteorological conditions.

The primary activities, such as fuel combustion that are responsible for NO_2_ emissions in the region are consistent throughout the year. This increases the impact of meteorological conditions on the pollutant levels of Delhi. This is further evident from similar contribution patterns during the PrM and PoM seasons. Since SO_2_ is a relatively short-lived gas, the contribution areas are smaller and limited to lower heights. The results from the WCWT analysis help us identify the industrial areas that play an essential role in the pollutant levels in Delhi, as clear hotspots are identifies over industrial regions of Punjab during PrM and PoM seasons.

It is imperative to state that the objective of this study is not to delineate accountability to any specific region or economic activity. The contribution hotspots are identified to understand the existing scenario better. These findings can help decision-makers isolate policies to reduce emission in a particular area. Such an approach will further prevent any detrimental effect on the economic growth of surrounding areas. The identification of such hotspots can assist in addressing air pollution at the source despite transboundary pollutant transportation in effect. The precise identification of such hotspots can further assist in establishing suitable administrative structures for potential application of environmental justice.

### Supplementary Information

Supplementary Information.

## Data Availability

All data sources and datasets are mentioned in the manuscripts and supplementary material.
