# Supplementary material for: Determining hotspots of gaseous criteria air pollutants in Delhi airshed and its association with stubble burning
Source: Sci Rep. 2024 Jan 10;14:986. doi: 10.1038/s41598-023-51140-x (PMC10782015; doi:10.1038/s41598-023-51140-x)
Supplement: Supplementary file 1 — Supplementary Information. [file 41598_2023_51140_MOESM1_ESM.pdf]

Supplementary Data

Supplementary Table

Supplementary Table 1: Details of criteria air pollutants retrieved from Sentinel-5P TROPOMI 1
Supplementary Table 2: CPCB Ground Station Data Collection Sites 1
Supplementary Table 3: Exceedance Days for CO (Sentinel 5P) from 2019 to 2022 3
Supplementary Table 4: Exceedance Days for NO_2 (Sentinel 5P) from 2019 to 2022 3
Supplementary Table 5: Exceedance Days for SO_2 (Sentinel 5P) from 2019 to 2022 4

Supplementary Table 1: Details of criteria air pollutants retrieved from Sentinel-5P TROPOMI

Pollutant	Atmospheric level of pollutant	Unit	From Date	To Date
**CO**	Total column CO	10^{-3} mol/m^2	01/01/2019	30/06/2022
**NO_2**	Tropospheric NO_2	10^{-5} mol/m^2	10/07/2018	30/06/2022
**SO_2**	Total column SO_2	Dobson Units	01/12/2018	30/06/2022

Supplementary Table 2: CPCB Ground Station Data Collection Sites

Site No.	Site	Agency	State	Lat	Lon
1	Alipur, Delhi - DPCC	Delhi Pollution Control Committee	Delhi	28.81533	77.15301
2	Anand Vihar, Delhi - DPCC	Delhi Pollution Control Committee	Delhi	28.64684	77.31603
3	Ashok Vihar, Delhi - DPCC	Delhi Pollution Control Committee	Delhi	28.69538	77.18167
4	Aya Nagar, Delhi - IMD	India Meteorological Department	Delhi	28.47069	77.10994
5	Bawana, Delhi - DPCC	Delhi Pollution Control Committee	Delhi	28.7762	77.05107
6	Burari Crossing, Delhi - IMD	India Meteorological Department	Delhi	28.72565	77.20116
7	Chandni Chowk, Delhi - IITM	Indian Institute Of Tropical Meteorology	Delhi	28.65676	77.22723
8	CRRI Mathura Road, Delhi - IMD	India Meteorological Department	Delhi	28.5512	77.27357
9	Dr. Karni Singh Shooting Range, Delhi - DPCC	Delhi Pollution Control Committee	Delhi	28.49857	77.26484
10	DTU, Delhi - CPCB	Central Pollution Control Board	Delhi	28.75005	77.11126
11	Dwarka-Sector 8, Delhi - DPCC	Delhi Pollution Control Committee	Delhi	28.57103	77.0719
12	East Arjun Nagar, Delhi - CPCB	Central Pollution Control Board	Delhi	28.65594	77.2949
13	IGI Airport (T3), Delhi - IMD	India Meteorological Department	Delhi	28.56278	77.11801
14	IHBAS, Dilshad Garden, Delhi - CPCB	Central Pollution Control Board	Delhi	28.68117	77.30252

15	ITO, Delhi - CPCB	Central Pollution Control Board	Delhi	28.62862	77.24106
16	Jahangirpuri, Delhi - DPCC	Delhi Pollution Control Committee	Delhi	28.73282	77.17063
17	Jawaharlal Nehru Stadium, Delhi - DPCC	Delhi Pollution Control Committee	Delhi	28.58028	77.23383
18	Lodhi Road, Delhi - IMD	India Meteorological Department	Delhi	28.59182	77.22731
19	Major Dhyan Chand National Stadium, Delhi - DPCC	Delhi Pollution Control Committee	Delhi	28.61128	77.23774
20	Mandir Marg, Delhi - DPCC	Delhi Pollution Control Committee	Delhi	28.63643	77.20107
21	Mundka, Delhi - DPCC	Delhi Pollution Control Committee	Delhi	28.68468	77.07657
22	Najafgarh, Delhi - DPCC	Delhi Pollution Control Committee	Delhi	28.57017	76.93376
23	Narela, Delhi - DPCC	Delhi Pollution Control Committee	Delhi	28.82284	77.10198
24	Nehru Nagar, Delhi - DPCC	Delhi Pollution Control Committee	Delhi	28.56789	77.25052
25	North Campus, DU, Delhi - IMD	India Meteorological Department	Delhi	28.65738	77.15854
26	NSIT Dwarka, Delhi - CPCB	Central Pollution Control Board	Delhi	28.60909	77.03254
27	Okhla Phase-2, Delhi - DPCC	Delhi Pollution Control Committee	Delhi	28.53079	77.27126
28	Patparganj, Delhi - DPCC	Delhi Pollution Control Committee	Delhi	28.62375	77.28721
29	Punjabi Bagh, Delhi - DPCC	Delhi Pollution Control Committee	Delhi	28.67405	77.13102
30	Pusa, Delhi - DPCC	Delhi Pollution Control Committee	Delhi	28.63965	77.14626
31	Pusa, Delhi - IMD	India Meteorological Department	Delhi	28.63704	77.17231
32	R K Puram, Delhi - DPCC	Delhi Pollution Control Committee	Delhi	28.56326	77.18694
33	Rohini, Delhi - DPCC	Delhi Pollution Control Committee	Delhi	28.73253	77.11992
34	Shadipur, Delhi - CPCB	Central Pollution Control Board	Delhi	28.65148	77.14731
35	Sirifort, Delhi - CPCB	Central Pollution Control Board	Delhi	28.55042	77.21594
36	Sonia Vihar, Delhi - DPCC	Delhi Pollution Control Committee	Delhi	28.71051	77.24949
37	Sri Aurobindo Marg, Delhi - DPCC	Delhi Pollution Control Committee	Delhi	28.53135	77.19016
38	Vivek Vihar, Delhi - DPCC	Delhi Pollution Control Committee	Delhi	28.67234	77.31526
39	Wazirpur, Delhi - DPCC	Delhi Pollution Control Committee	Delhi	28.69979	77.16545

Supplementary Table 3: Exceedance Days for CO (Sentinel 5P) from 2019 to 2022

	2019	2020	2021	2022
**Day Availability (%)**	84.93	89.04	88.22	88.22
**Mean** (10^{-3} mol/m^2)	41.07	41.05	41.69	40.87
**Standard Deviation** (±10^{-3} mol/m^2)	6.05	6.03	6.36	5.89
**% Days Exceedance for Mean**				
Annual	43.55	45.23	41.30	43.79
Winter	11.29	15.69	17.08	13.66
Pre-Monsoon	12.26	9.85	9.94	15.22
Monsoon	10.00	5.54	4.66	4.97
Post-Monsoon	10.00	14.15	9.63	9.94
**% Days Exceedance for 1st Standard Deviation**				
Annual	12.26	11.08	16.77	13.04
Winter	3.87	1.85	6.21	2.48
Pre-Monsoon	2.26	0.62	3.42	5.59
Monsoon	1.61	0.62	0.00	0.93
Post-Monsoon	4.52	8.00	7.14	4.04

Supplementary Table 4: Exceedance Days for NO_2 (Sentinel 5P) from 2019 to 2022

	2019	2020	2021	2022
**Day Availability (%)**	84.66	86.58	87.95	87.40
**Mean** (10^{-5} mol/m^2)	11.02	8.59	11.75	11.84
**Standard Deviation** (±10^{-5} mol/m^2)	7.58	5.04	8.38	6.28
**% Days Exceedance for Mean**				
Annual	32.36	38.92	29.60	33.86
Winter	14.89	20.89	16.20	16.30
Pre-Monsoon	6.80	3.80	3.43	8.46
Monsoon	5.18	3.80	1.56	2.19
Post-Monsoon	5.50	10.44	8.41	6.90
**% Days Exceedance for 1st Standard Deviation**				
Annual	10.00	11.38	10.56	13.35
Winter	8.39	8.31	7.14	9.63
Pre-Monsoon	0.00	1.23	0.62	1.24
Monsoon	0.00	0.00	0.00	0.00
Post-Monsoon	1.61	1.85	2.80	2.48

Supplementary Table 5: Exceedance Days for SO_2 (Sentinel 5P) from 2019 to 2022

	2019	2020	2021	2022
**Day Availability (%)**	87.12	86.85	84.66	84.38
**Mean** (DU)	0.82	0.87	0.82	0.88
**Standard Deviation** (± DU)	0.58	0.46	0.49	0.58
**% Days Exceedance for Mean**				
Annual	33.33	37.85	38.19	34.09
Winter	15.09	16.09	13.92	12.34
Pre-Monsoon	9.43	9.46	12.30	9.74
Monsoon	5.97	5.68	4.85	5.52
Post-Monsoon	2.83	6.62	7.12	6.49
**% Days Exceedance for 1st Standard Deviation**				
Annual	9.68	12.00	10.25	7.45
Winter	7.42	7.38	4.35	4.04
Pre-Monsoon	1.29	2.15	2.48	0.62
Monsoon	0.32	0.31	0.93	0.62
Post-Monsoon	0.65	2.15	2.48	2.17

Supplementary Figures

Supplementary Figure S 1: (a) The study area location of National Capital Territory and National Capital Region; (b) On ground pictures of SB in Punjab 2022 (Source: Author) 7
Supplementary Figure S2: Flowchart of adopted Methodology 8
Supplementary Figure S3: Daily Carbon Monoxide levels in Delhi NCR for 2019, 2020 and 2021 festive seasons 9
Supplementary Figure S4: Weekly Carbon Monoxide Trends in Delhi and Mixing Height in Punjab from 2018 December to 2019 November 10
Supplementary Figure S5: Weekly Carbon Monoxide Trends in Delhi and Temperature in Punjab from 2018 December to 2019 November 11
Supplementary Figure S6: Weekly Carbon Monoxide Trends in Delhi and Net Solar Radiation in Punjab from 2018 December to 2019 November 12
Supplementary Figure S7: Weekly NO_2 trends in Delhi NCT and air temperature in the study area (December 2018 to November 2019) 13
Supplementary Figure S8: Weekly Nitrogen Dioxide Trends in Delhi and Mixing Height from 2018 December to 2019 November 14
Supplementary Figure S9: Weekly Nitrogen Dioxide Trends in Delhi and Net Solar Radiation from 2018 December to 2019 November 15
Supplementary Figure S10: Weekly SO_2 trends in Delhi NCT and net solar radiation (NSR) in the study area (December 2018 to November 2019) 16
Supplementary Figure S11: Weekly Sulphur Dioxide Trends in Delhi and Mixing Height from 2018 December to 2019 November 17
Supplementary Figure S12: Weekly Sulphur Dioxide Trends in Delhi and Temperature from 2018 December to 2019 November 18
Supplementary Figure S13: Annual WCWT results for CO using CAMS and CPCB data at varying heights and composite result 19
Supplementary Figure S14: Winter WCWT results for CO using CAMS and CPCB data at varying heights and composite result 20
Supplementary Figure S15: PrM WCWT results for CO using CAMS and CPCB data at varying heights and composite result 21
Supplementary Figure S16: Monsoon WCWT results for CO using CAMS and CPCB data at varying heights and composite result 22
Supplementary Figure S17: PoM WCWT results for CO using CAMS and CPCB data at varying heights and composite result 23
Supplementary Figure S18: Fire Density Maps for different seasons over India 24
Supplementary Figure S19: Annual WCWT results for NO2 using CAMS and CPCB data at varying heights and composite result 25
Supplementary Figure S20 Winter WCWT results for NO2 using CAMS and CPCB data at varying heights and composite result 26
Supplementary Figure S21: PrM WCWT results for NO2 using CAMS and CPCB data at varying heights and composite result 27
Supplementary Figure S22: Monsoon WCWT results for NO2 using CAMS and CPCB data at varying heights and composite result 28
Supplementary Figure S23: Post-Monsoon WCWT results for NO2 using CAMS and CPCB data at varying heights and composite result 29
Supplementary Figure S24: Annual WCWT results for SO2 using CAMS and CPCB data at varying heights and composite result 30

Supplementary Figure S25: Winter WCWT results for SO2 using CAMS and CPCB data at varying heights and composite result 31
Supplementary Figure S26: PrM WCWT results for SO2 using CAMS and CPCB data at varying heights and composite result 32
Supplementary Figure S27: Monsoon WCWT results for SO2 using CAMS and CPCB data at varying heights and composite result 33
Supplementary Figure S28: PoM WCWT results for SO2 using CAMS and CPCB data at varying heights and composite result 34
Supplementary Figure S29: Annual and Seasonal average Temperature (MODIS), Mixing Height (ERA 5) and Wind Profile (ERA 5) for 2019 35

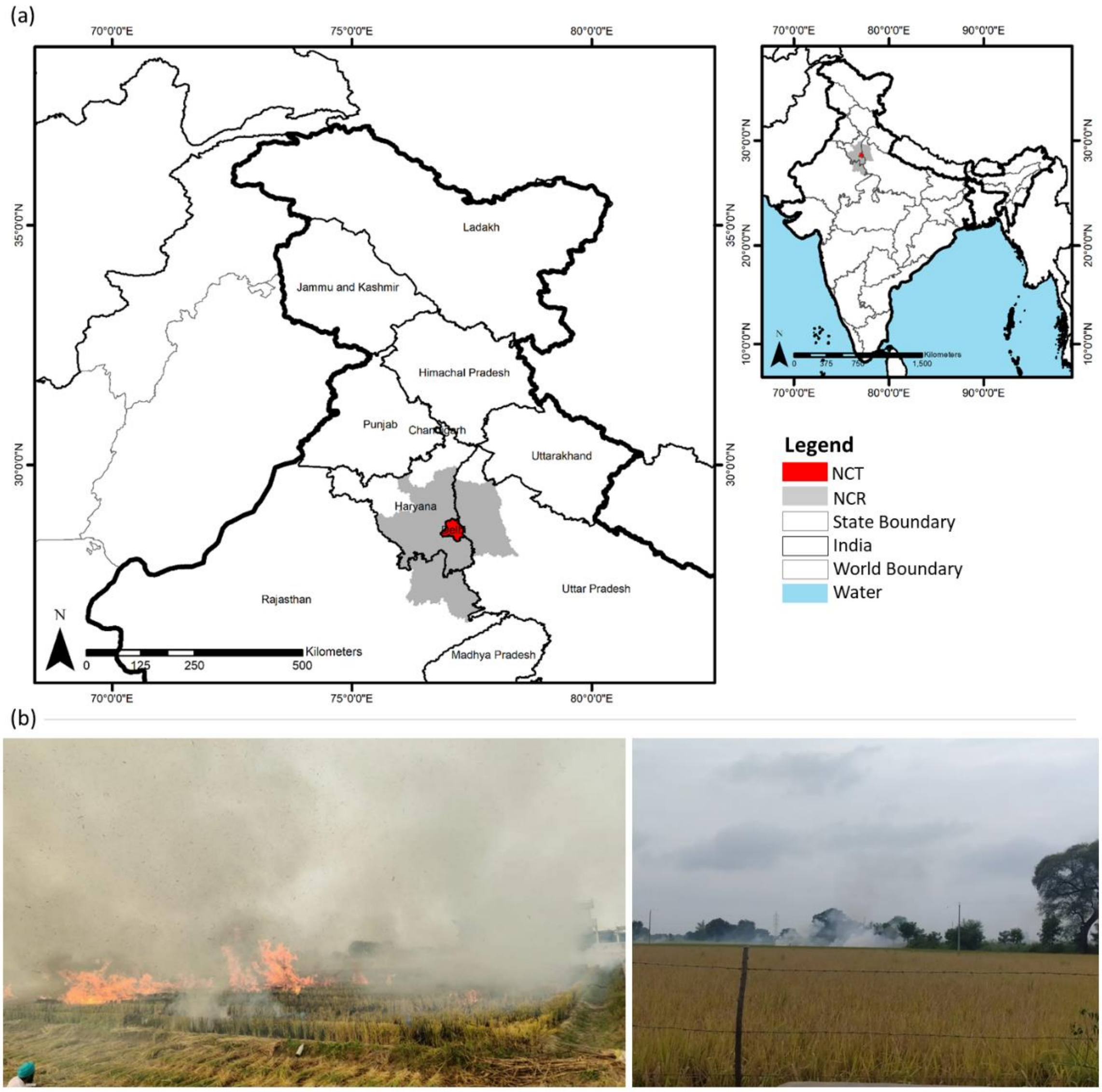

Supplementary Figure S 1: (a) The study area location of National Capital Territory and National Capital Region; (b) On ground pictures of SB in Punjab 2022 (Source: Author)

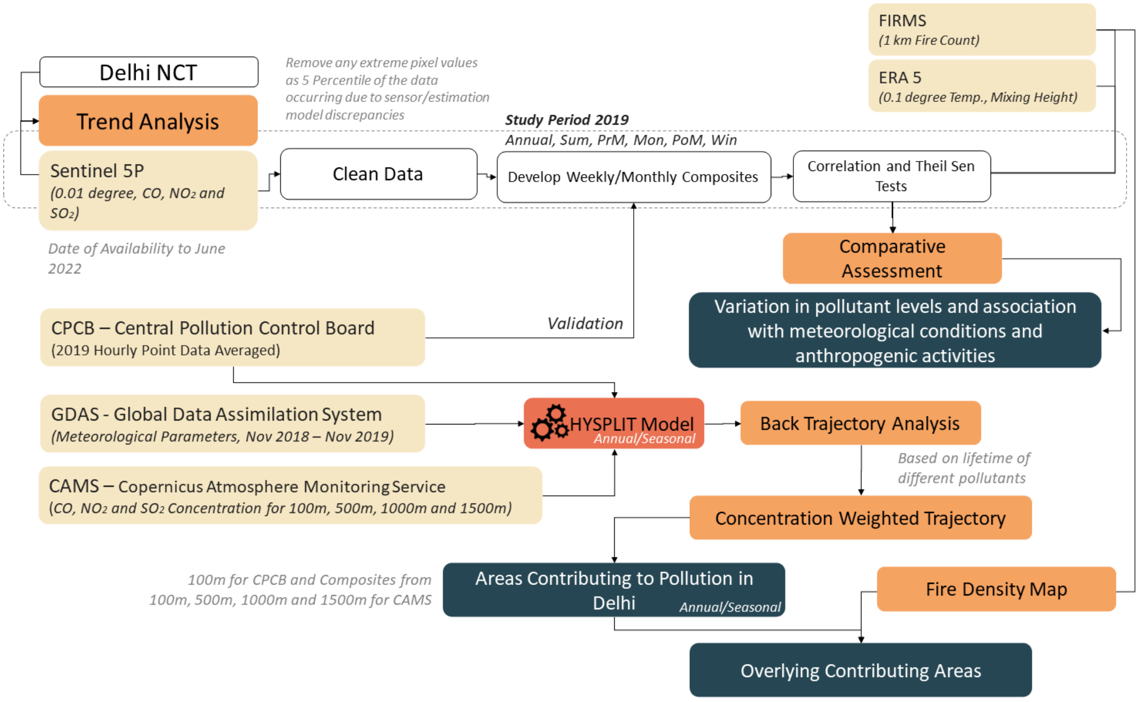

Supplementary Figure S2: Flowchart of adopted Methodology

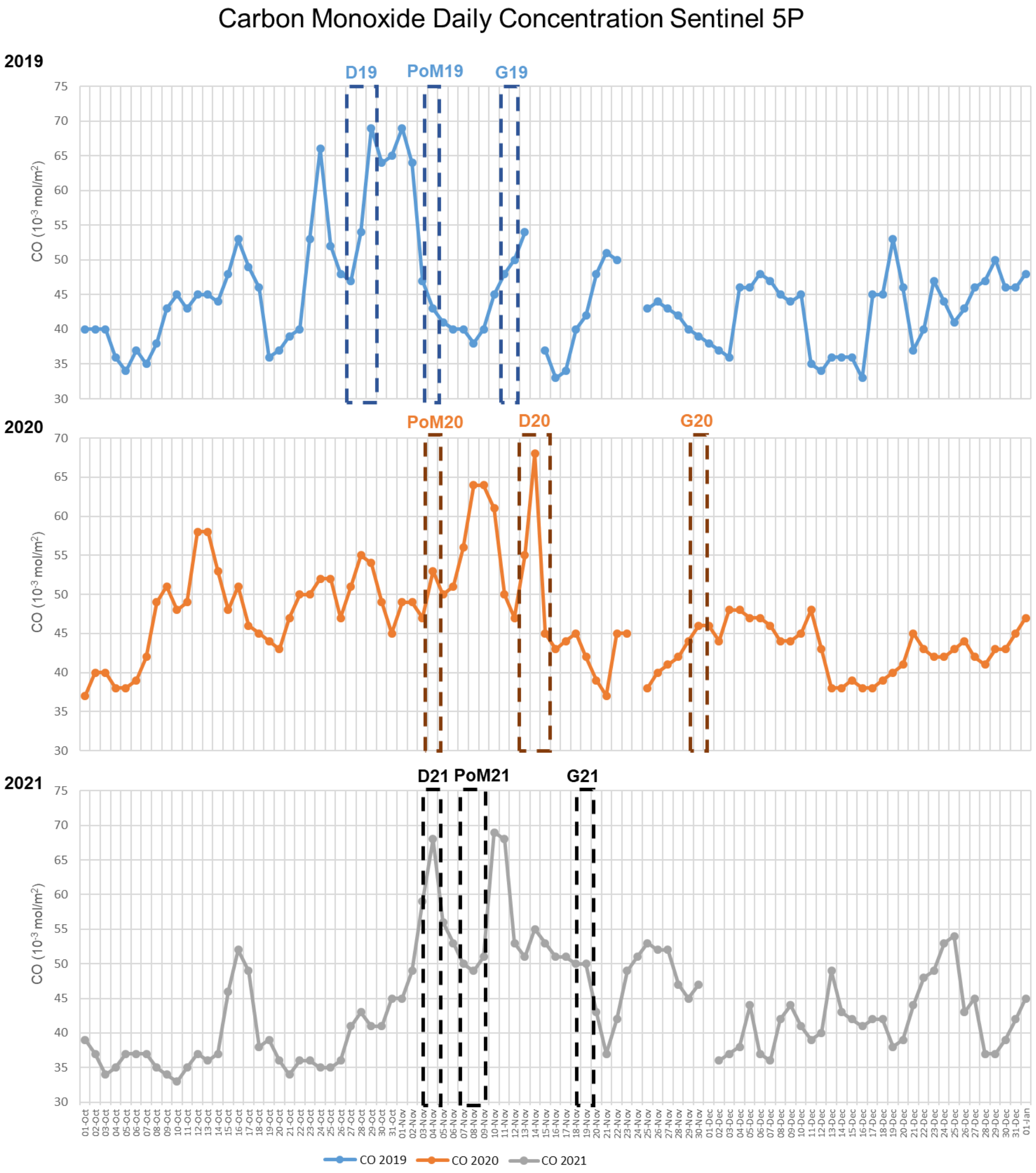

D19: Diwali 27th October 2019
G19: Gurupurab 12th November 2019
D20: Diwali 14th November 2020
G20: Gurupurab 30th November 2020
D21: Diwali 4th November 2021
G20: Gurupurab 19th November 2021

PoM19: Post-Monsoon Fire Peak 2019
PoM20: Post-Monsoon Fire Peak 2020
PoM21: Post-Monsoon Fire Peak 2021

Supplementary Figure S3: Daily Carbon Monoxide levels in Delhi NCR for 2019, 2020 and 2021 festive seasons

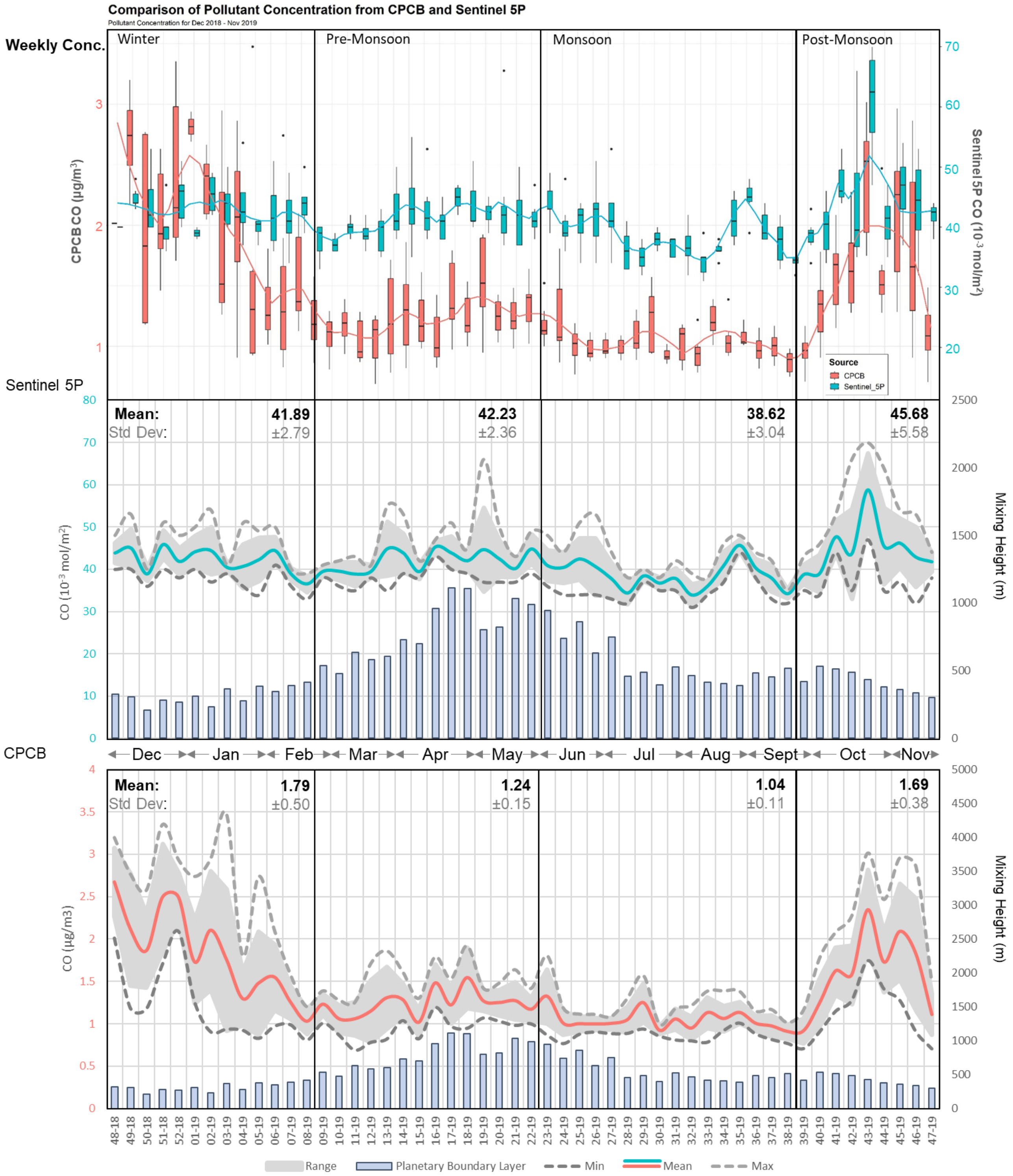

Supplementary Figure S4: Weekly Carbon Monoxide Trends in Delhi and Mixing Height in Punjab from 2018 December to 2019 November

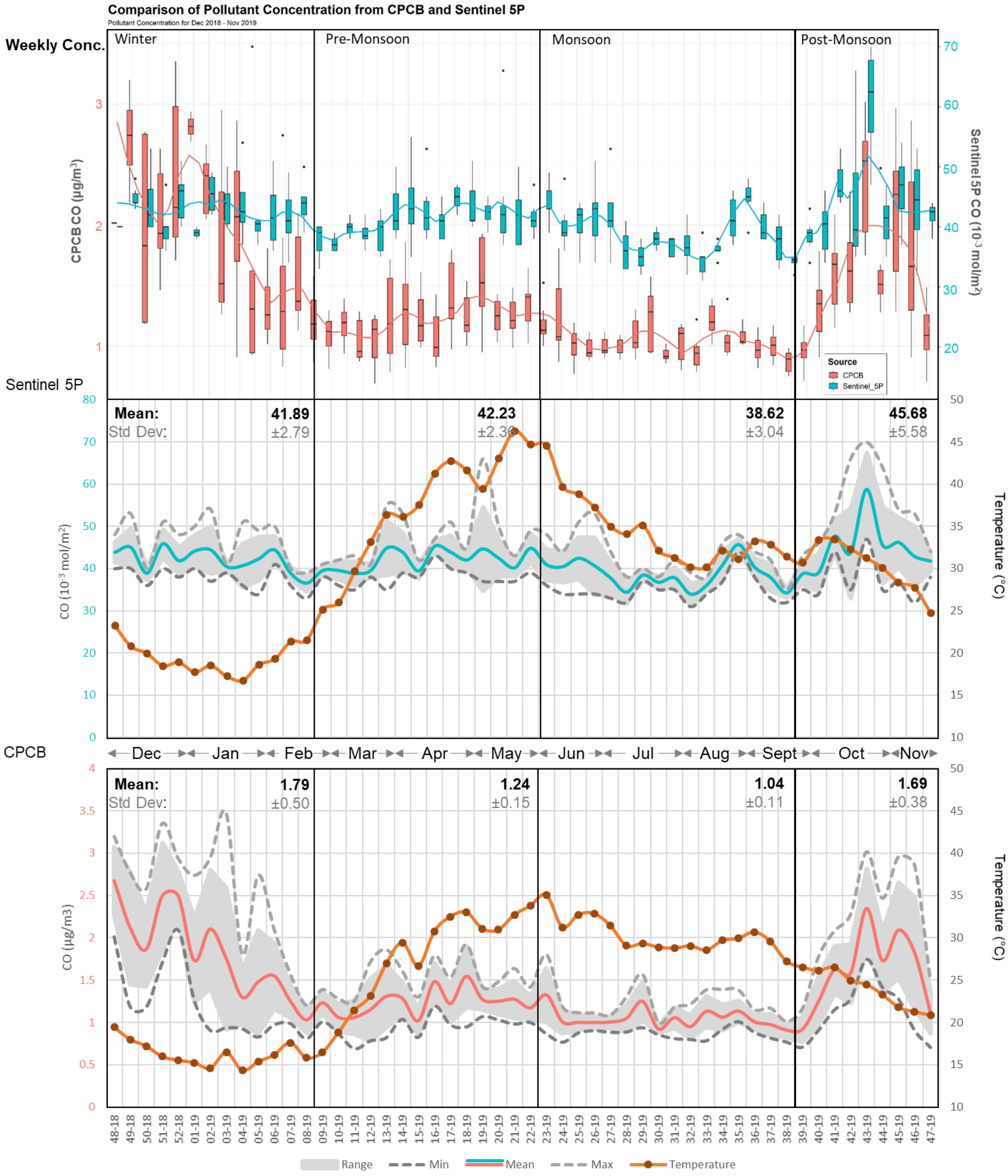

Supplementary Figure S5: Weekly Carbon Monoxide Trends in Delhi and Temperature in Punjab from 2018 December to 2019 November

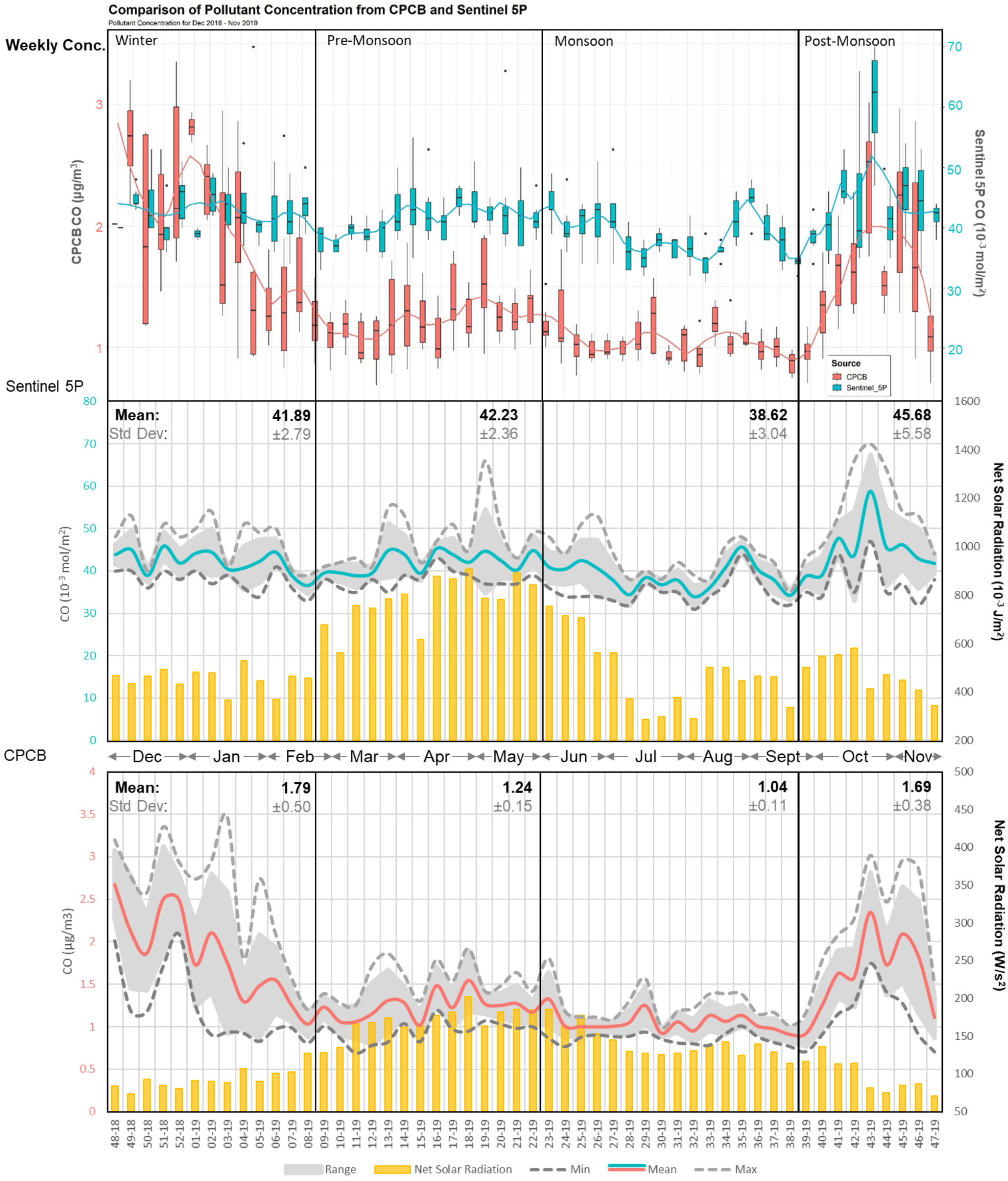

Supplementary Figure S6: Weekly Carbon Monoxide Trends in Delhi and Net Solar Radiation in Punjab from 2018 December to 2019 November

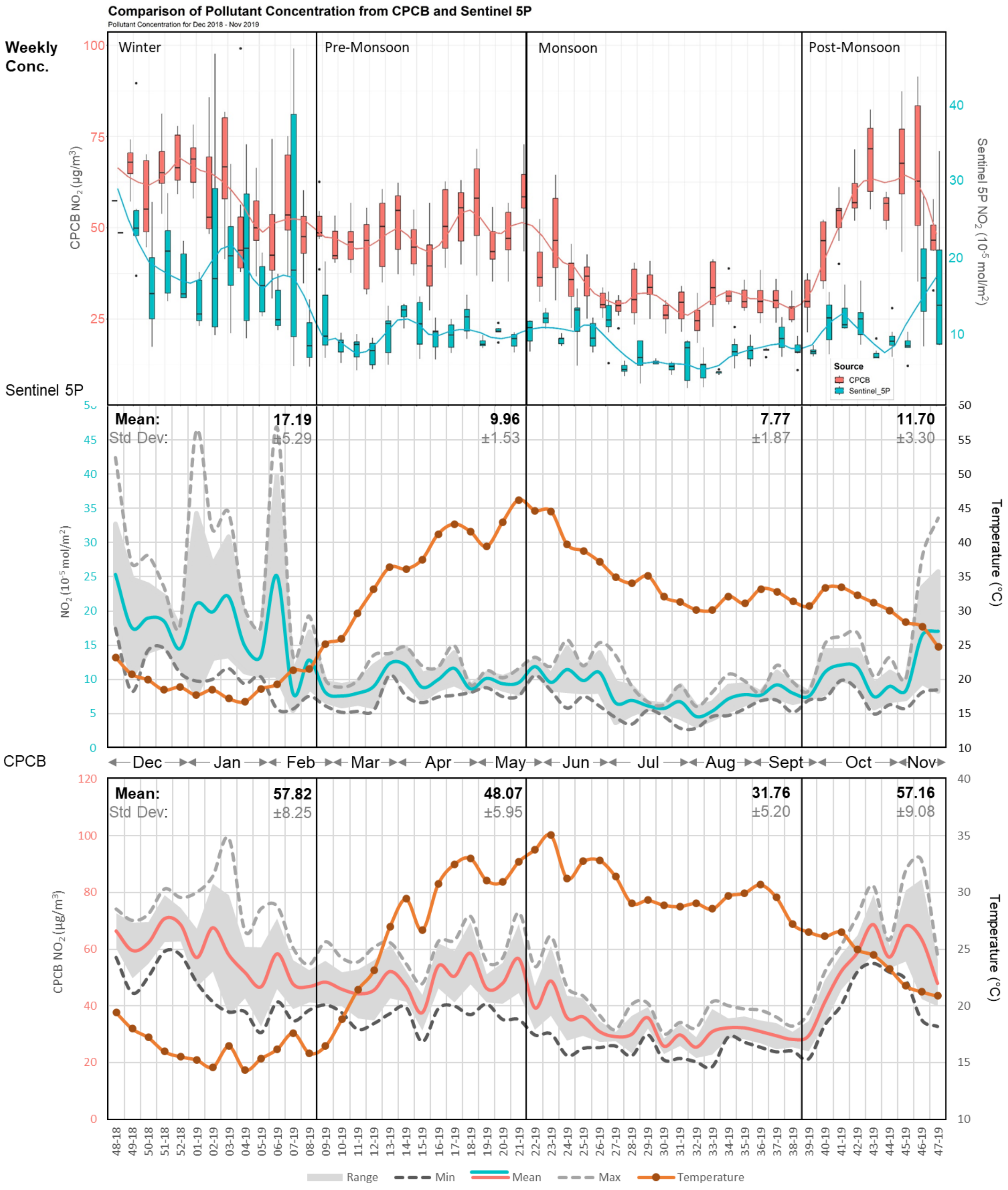

Supplementary Figure S7: Weekly NO_2 trends in Delhi NCT and air temperature in the study area (December 2018 to November 2019)

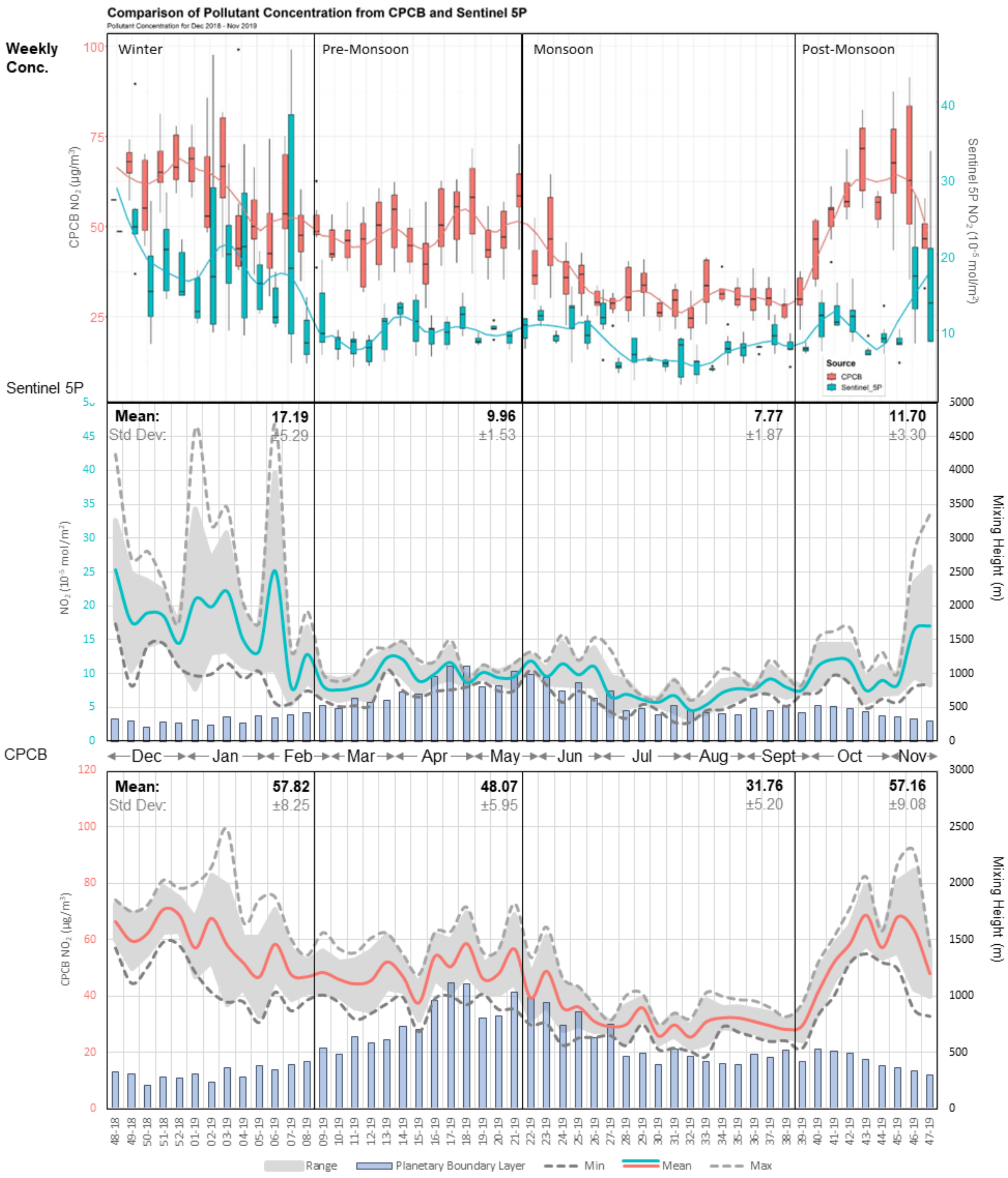

Supplementary Figure S8: Weekly Nitrogen Dioxide Trends in Delhi and Mixing Height from 2018 December to 2019 November

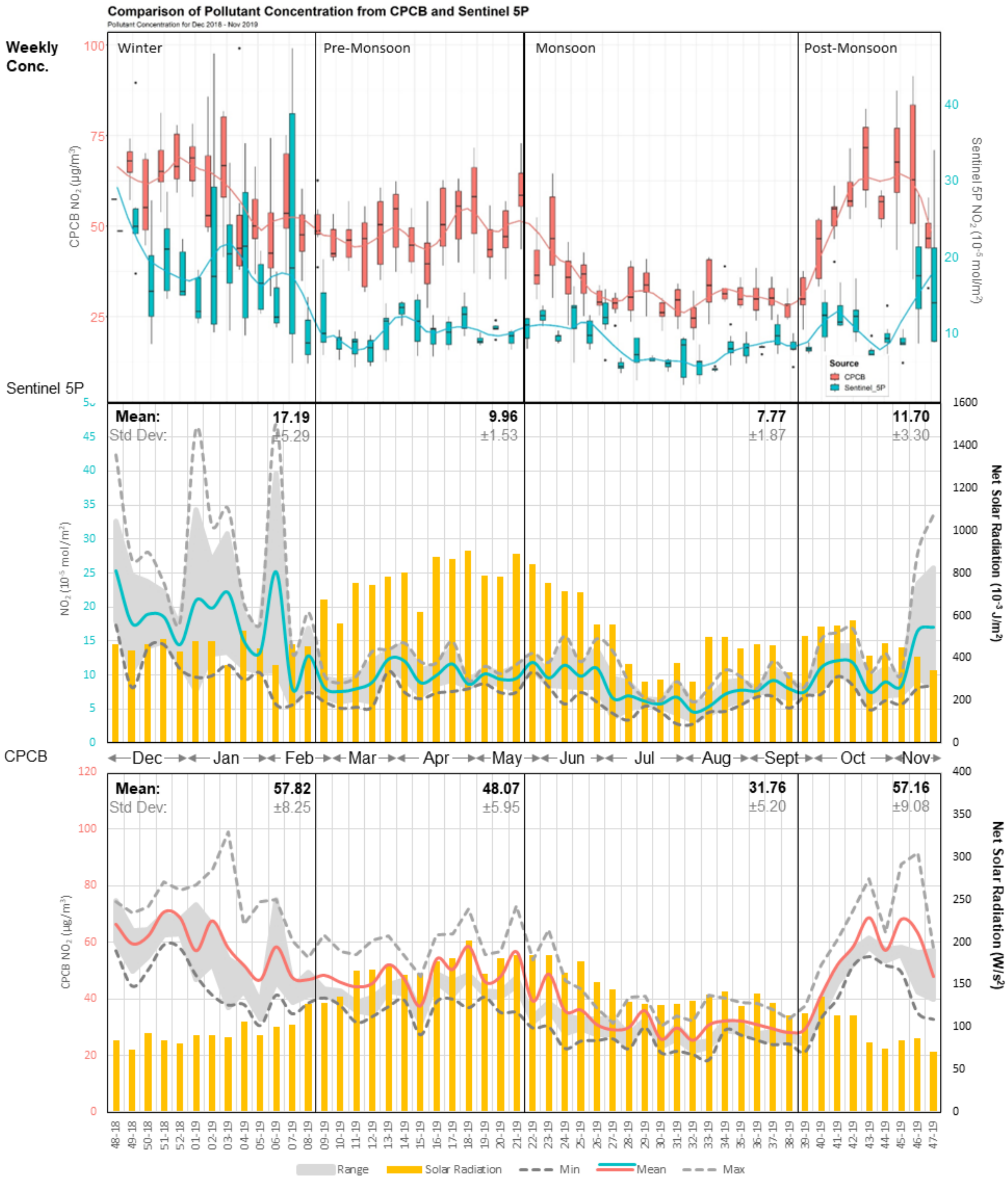

Supplementary Figure S9: Weekly Nitrogen Dioxide Trends in Delhi and Net Solar Radiation from 2018 December to 2019 November

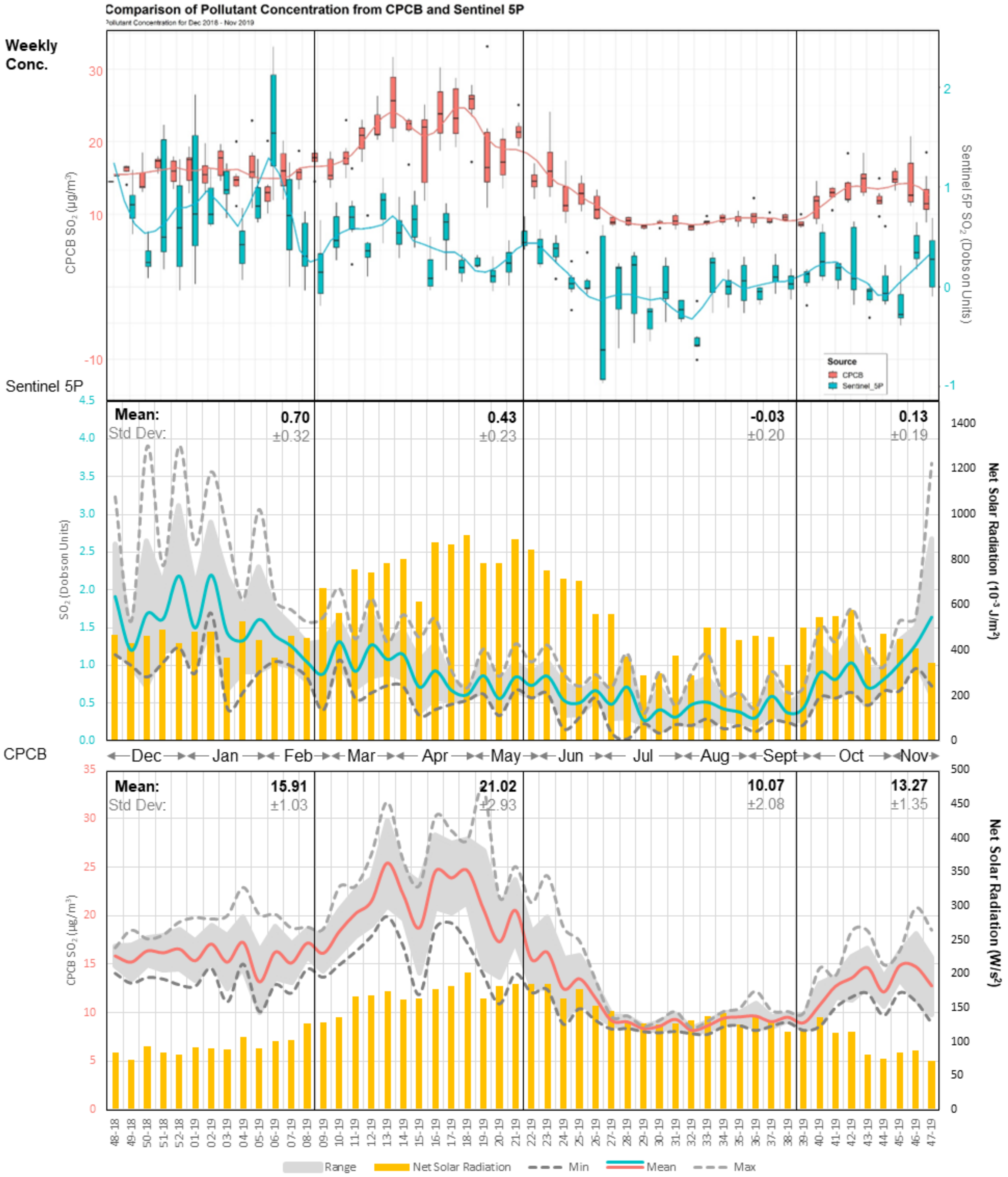

Supplementary Figure S10: Weekly SO_2 trends in Delhi NCT and net solar radiation (NSR) in the study area (December 2018 to November 2019)

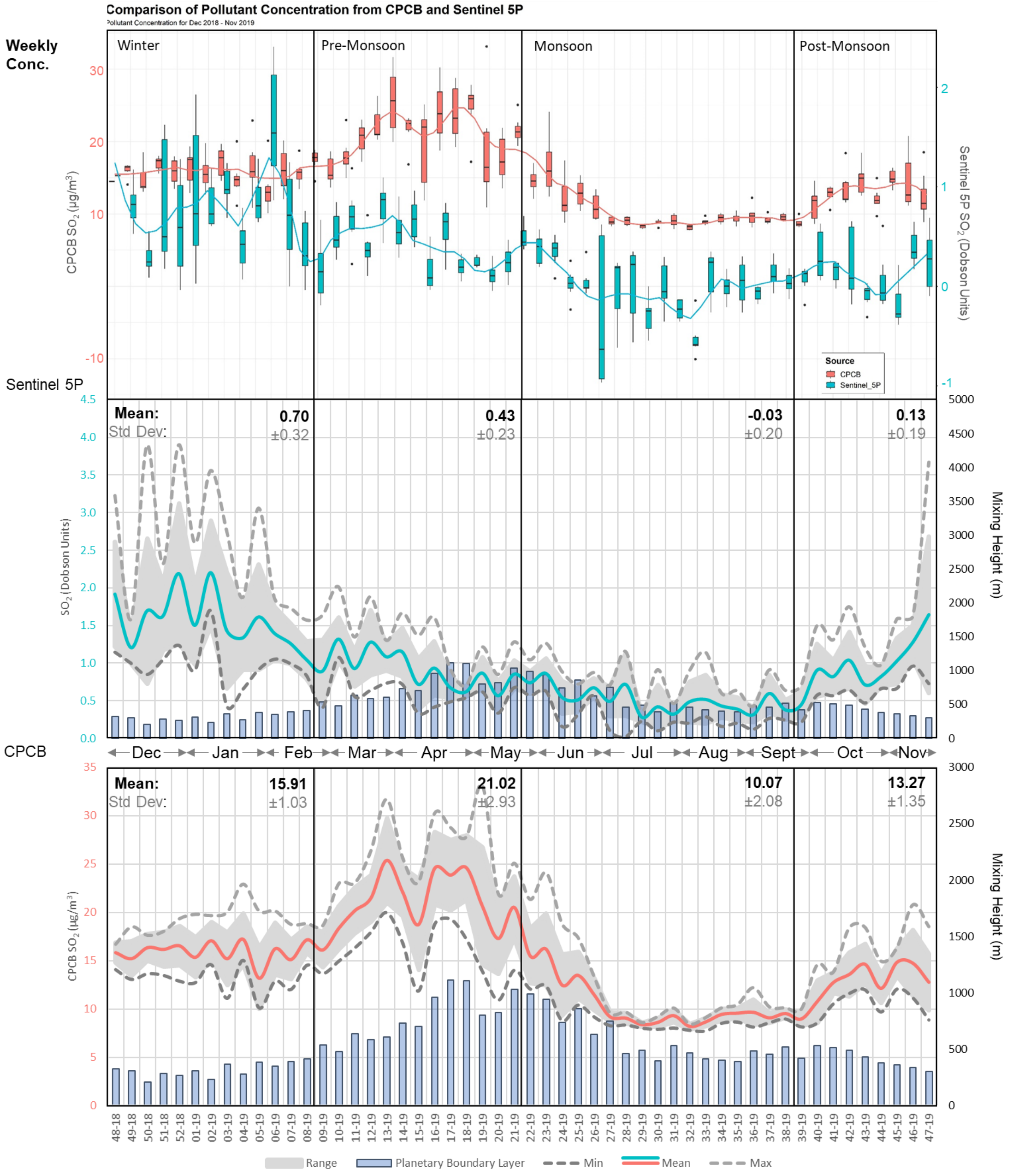

Supplementary Figure S11: Weekly Sulphur Dioxide Trends in Delhi and Mixing Height from 2018 December to 2019 November

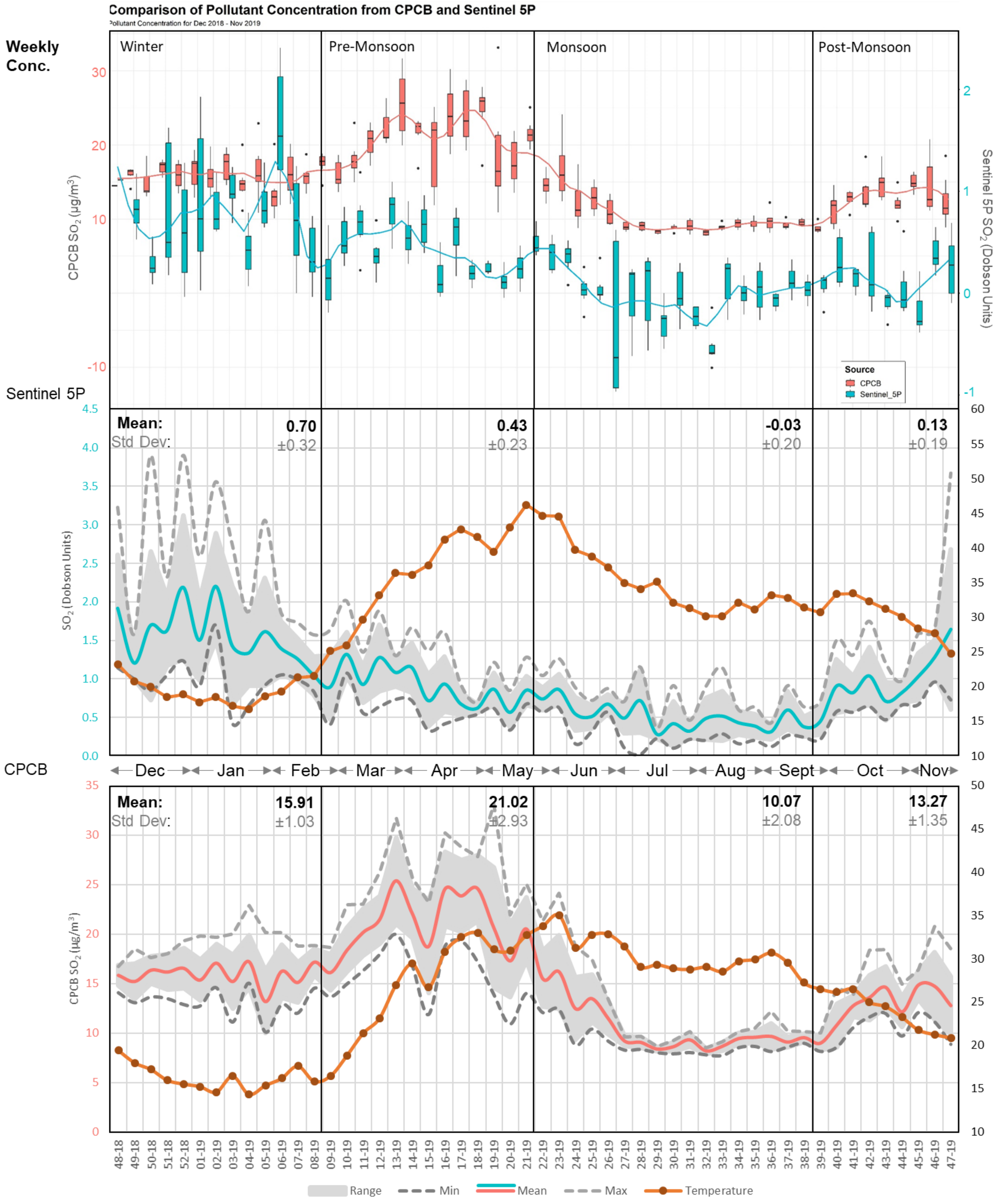

Supplementary Figure S12: Weekly Sulphur Dioxide Trends in Delhi and Temperature from 2018 December to 2019 November

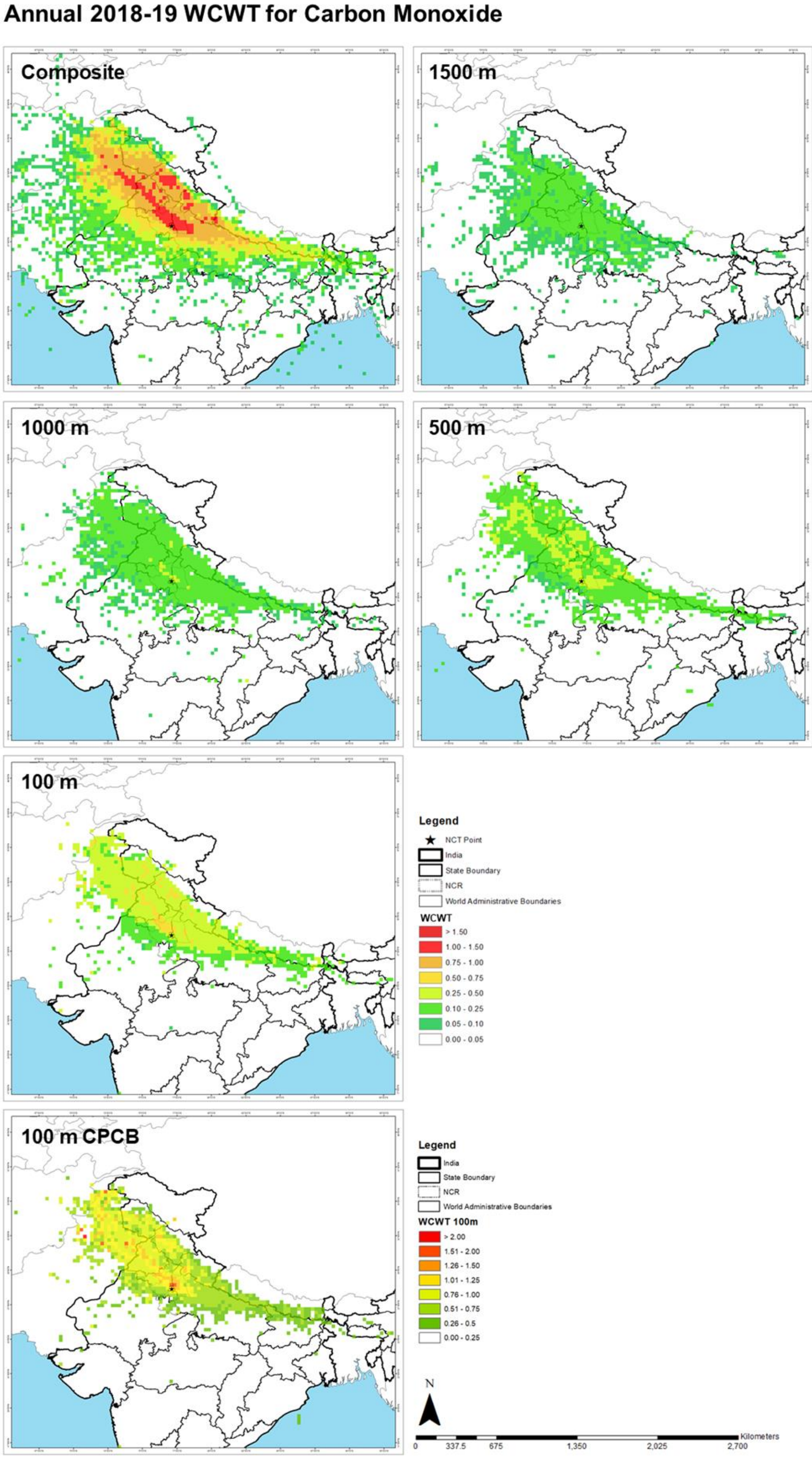

Supplementary Figure S13: Annual WCWT results for CO using CAMS and CPCB data at varying heights and composite result

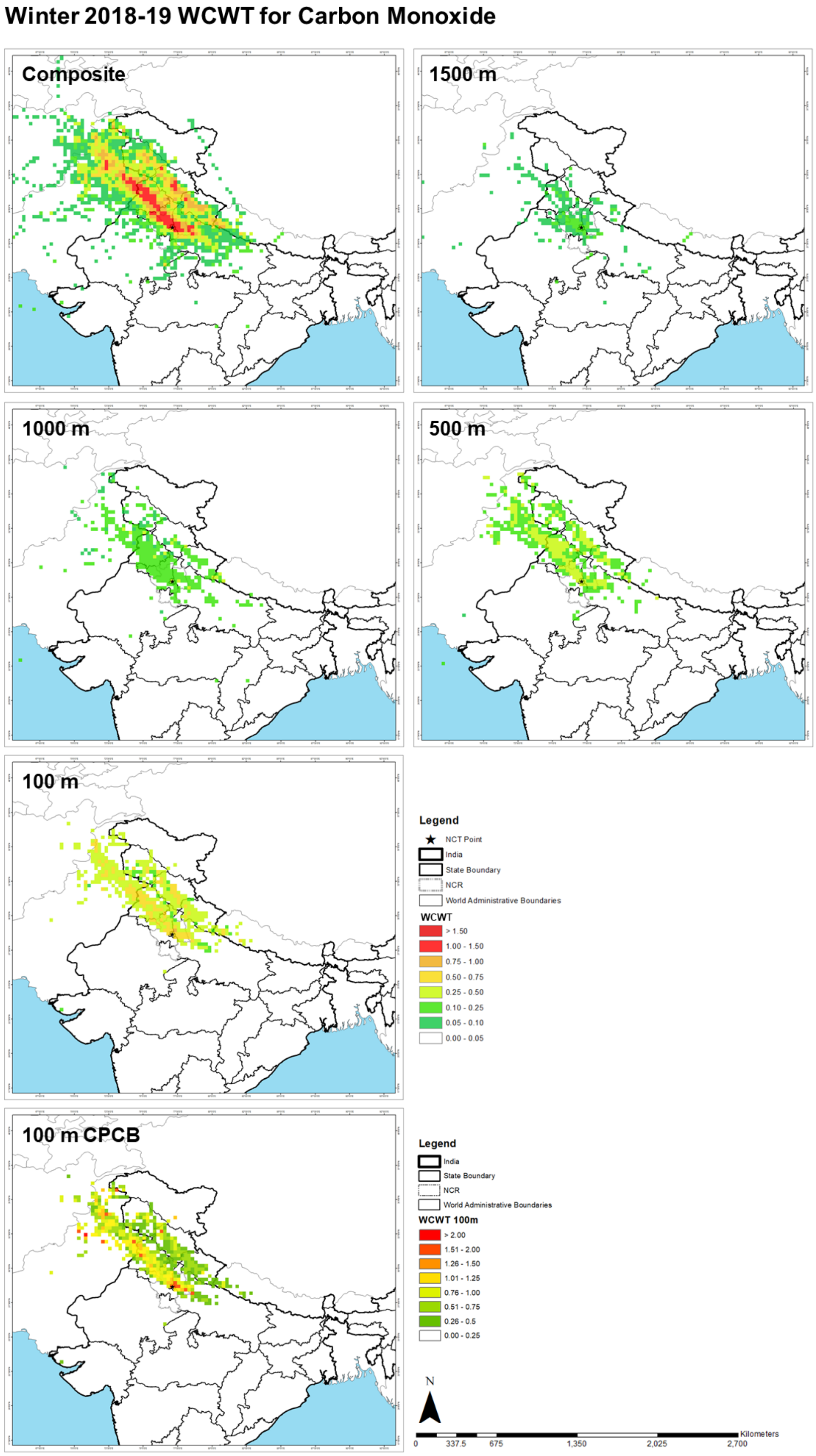

Supplementary Figure S14: Winter WCWT results for CO using CAMS and CPCB data at varying heights and composite result

Pre-Monsoon 2019 WCWT for Carbon Monoxide

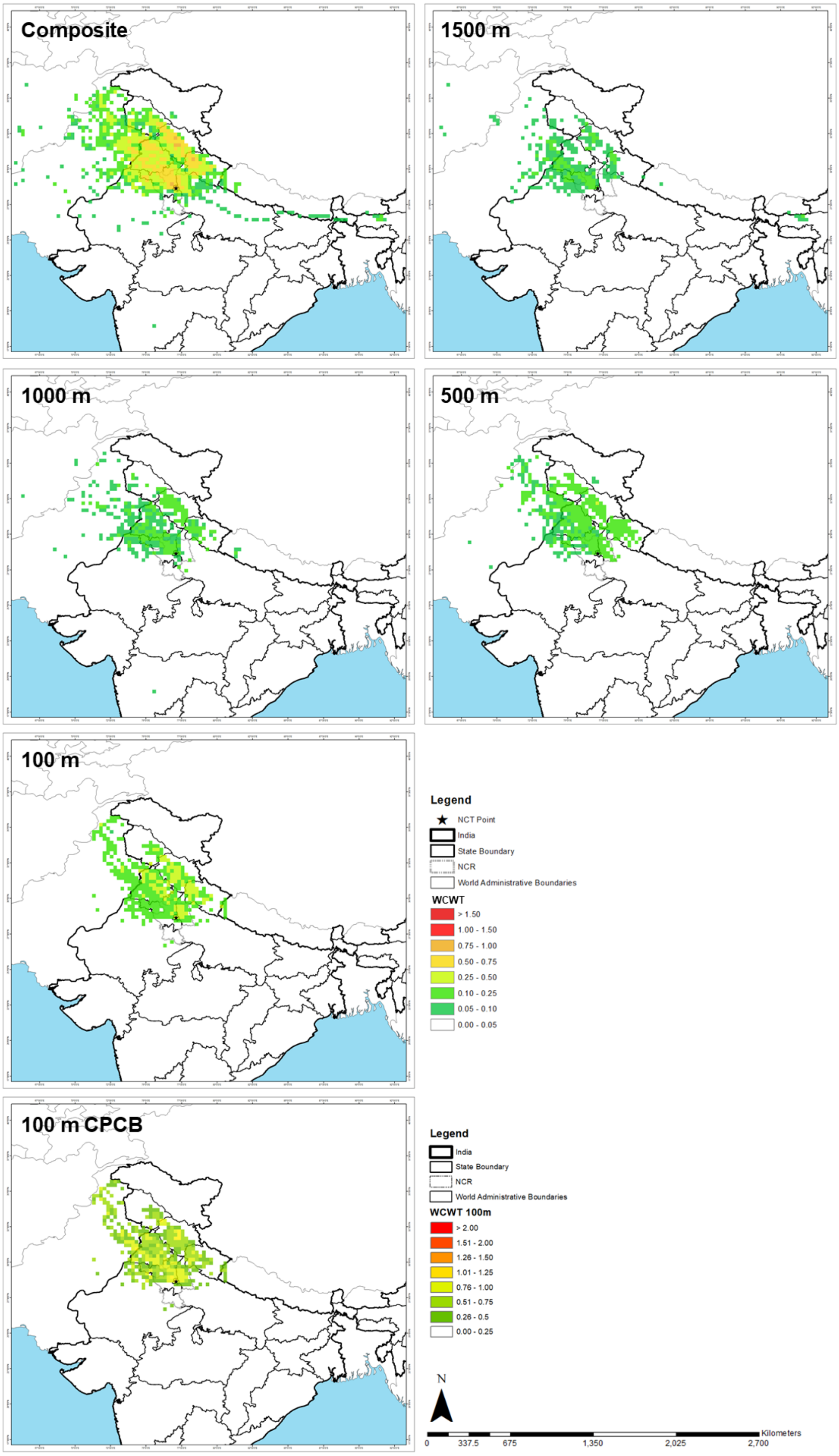

Supplementary Figure S15: PrM WCWT results for CO using CAMS and CPCB data at varying heights and composite result

Monsoon 2019 WCWT for Carbon Monoxide

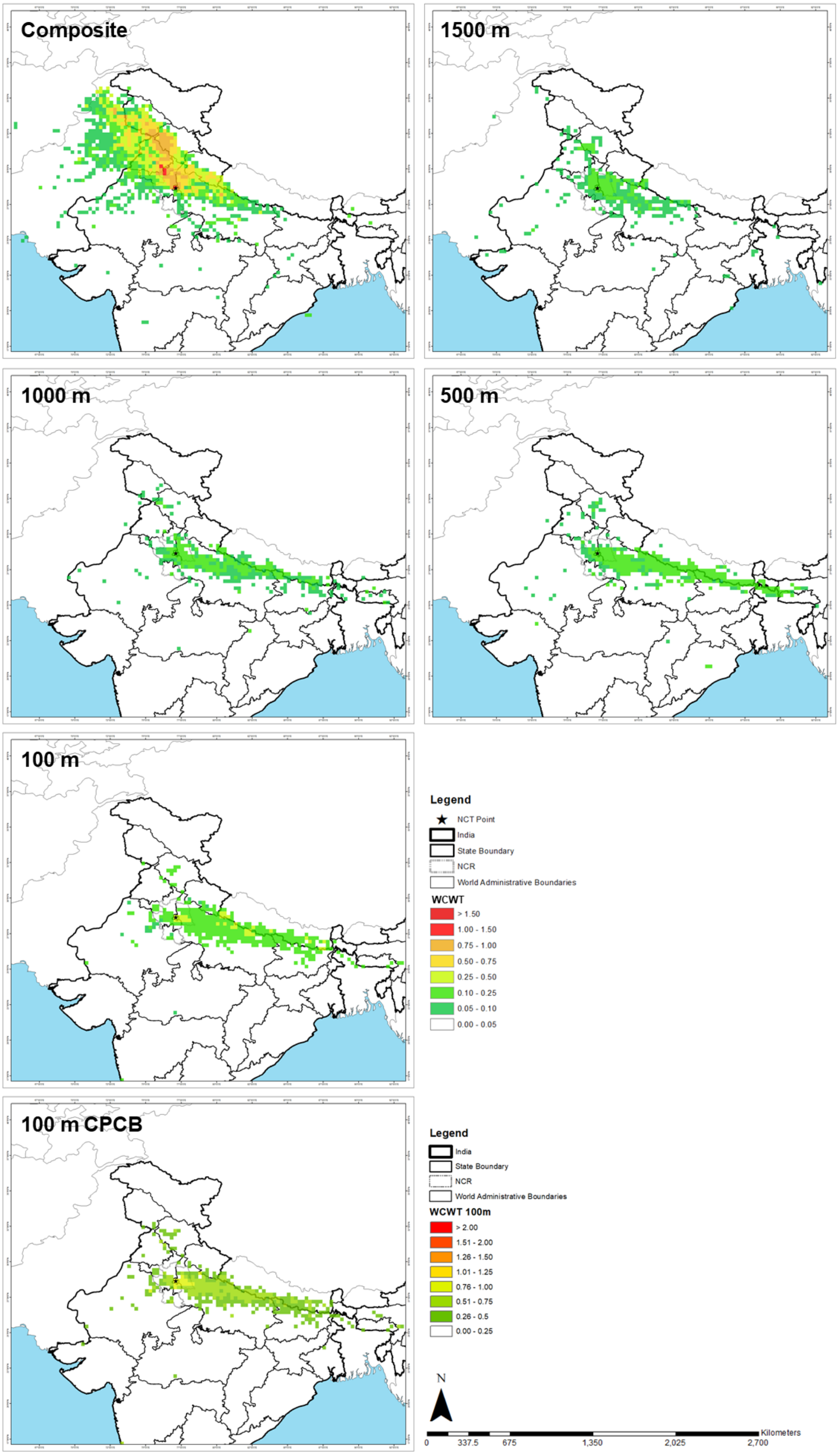

Supplementary Figure S16: Monsoon WCWT results for CO using CAMS and CPCB data at varying heights and composite result

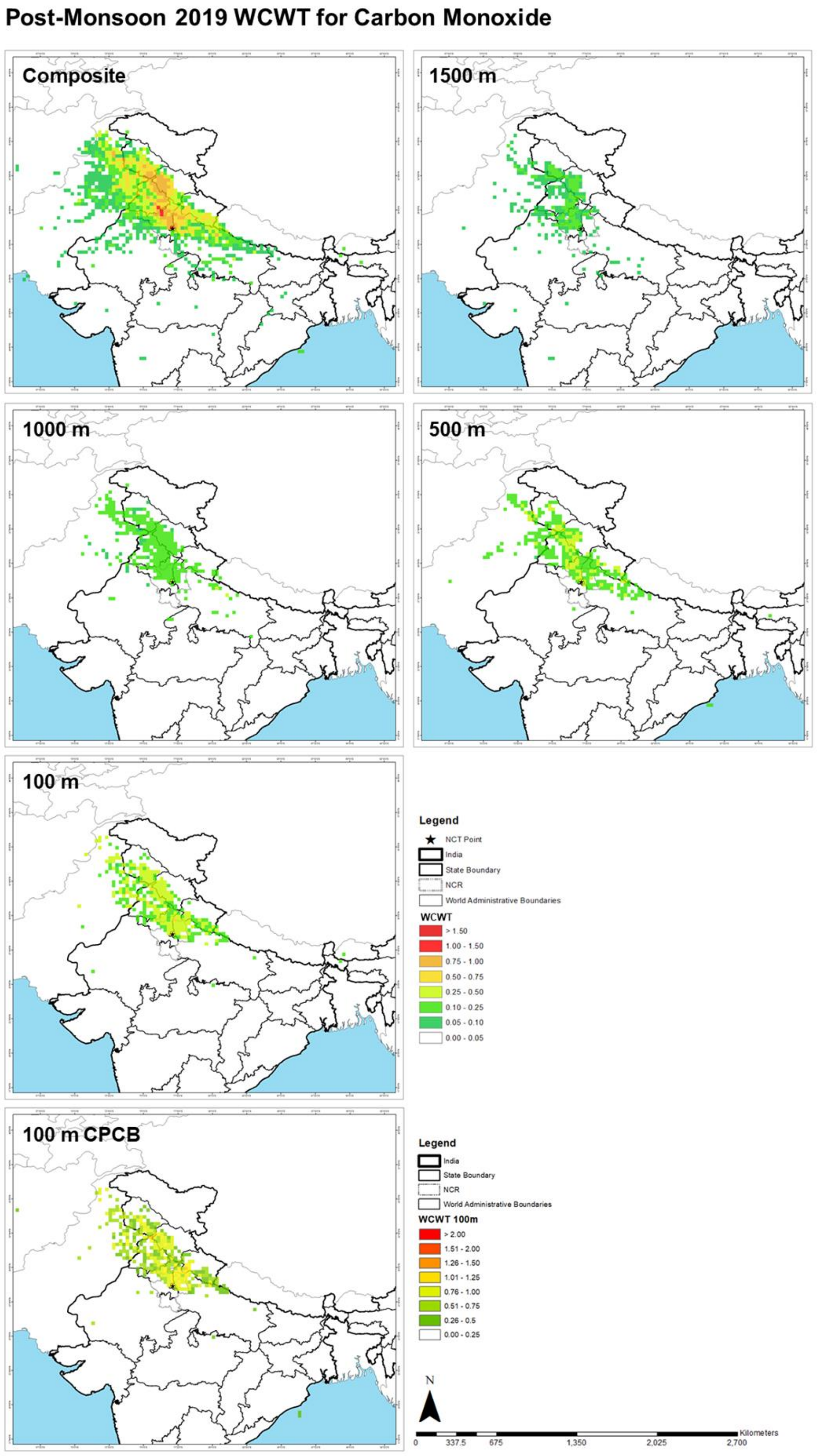

Supplementary Figure S17: PoM WCWT results for CO using CAMS and CPCB data at varying heights and composite result

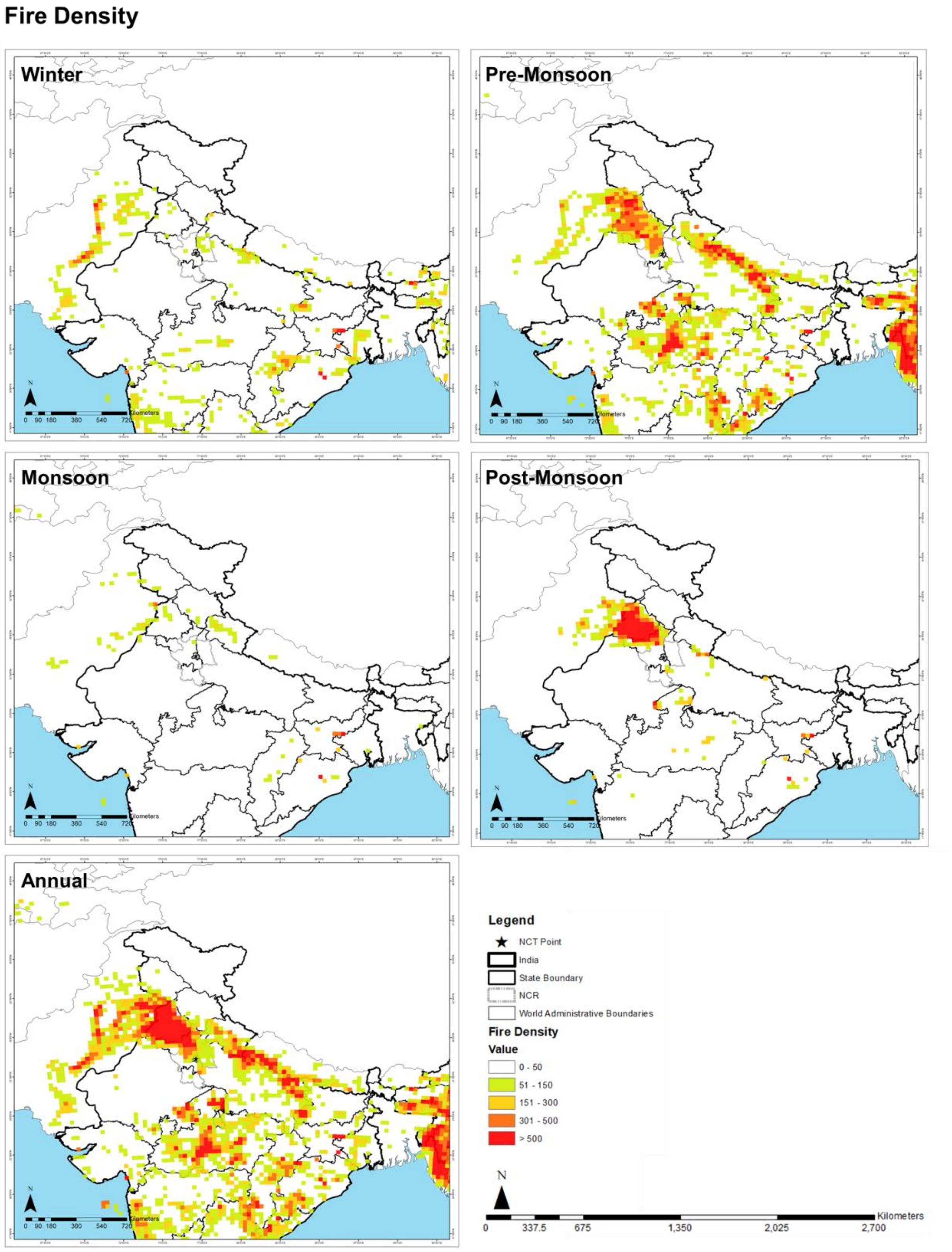

Supplementary Figure S18: Fire Density Maps for different seasons over India

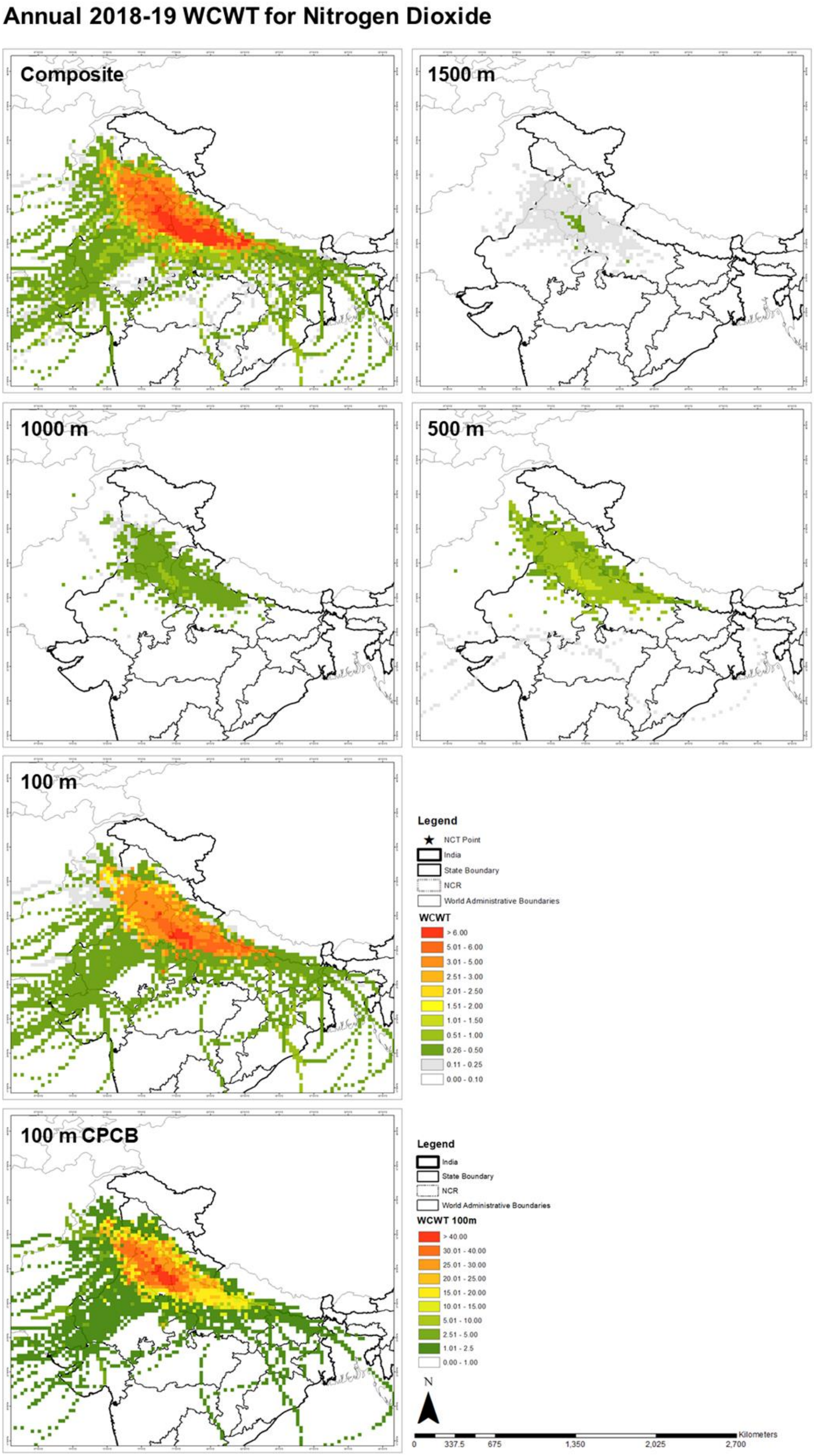

Supplementary Figure S19: Annual WCWT results for NO2 using CAMS and CPCB data at varying heights and composite result

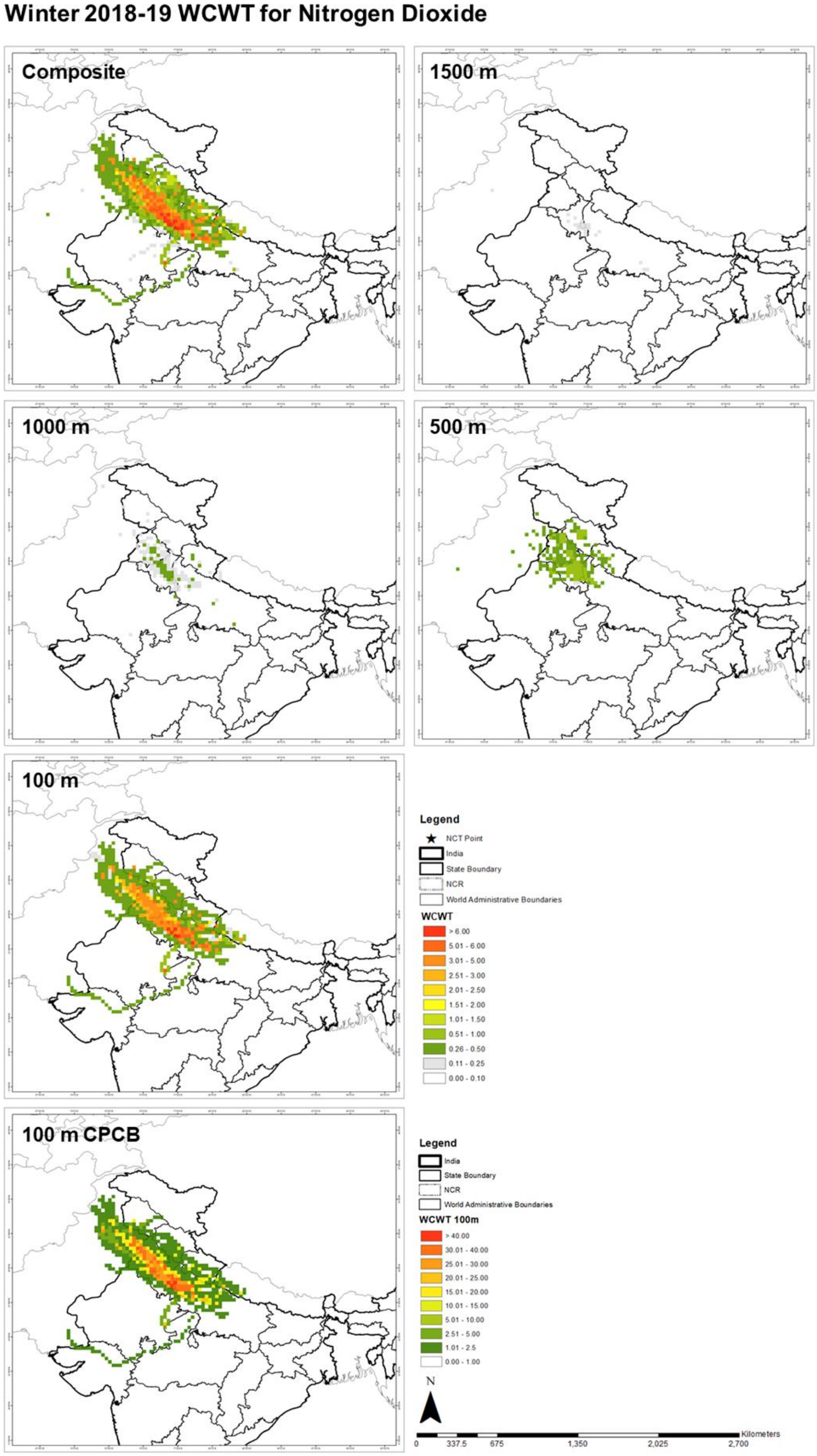

Supplementary Figure S20 Winter WCWT results for NO2 using CAMS and CPCB data at varying heights and composite result

Pre-Monsoon 2019 WCWT for Nitrogen Dioxide

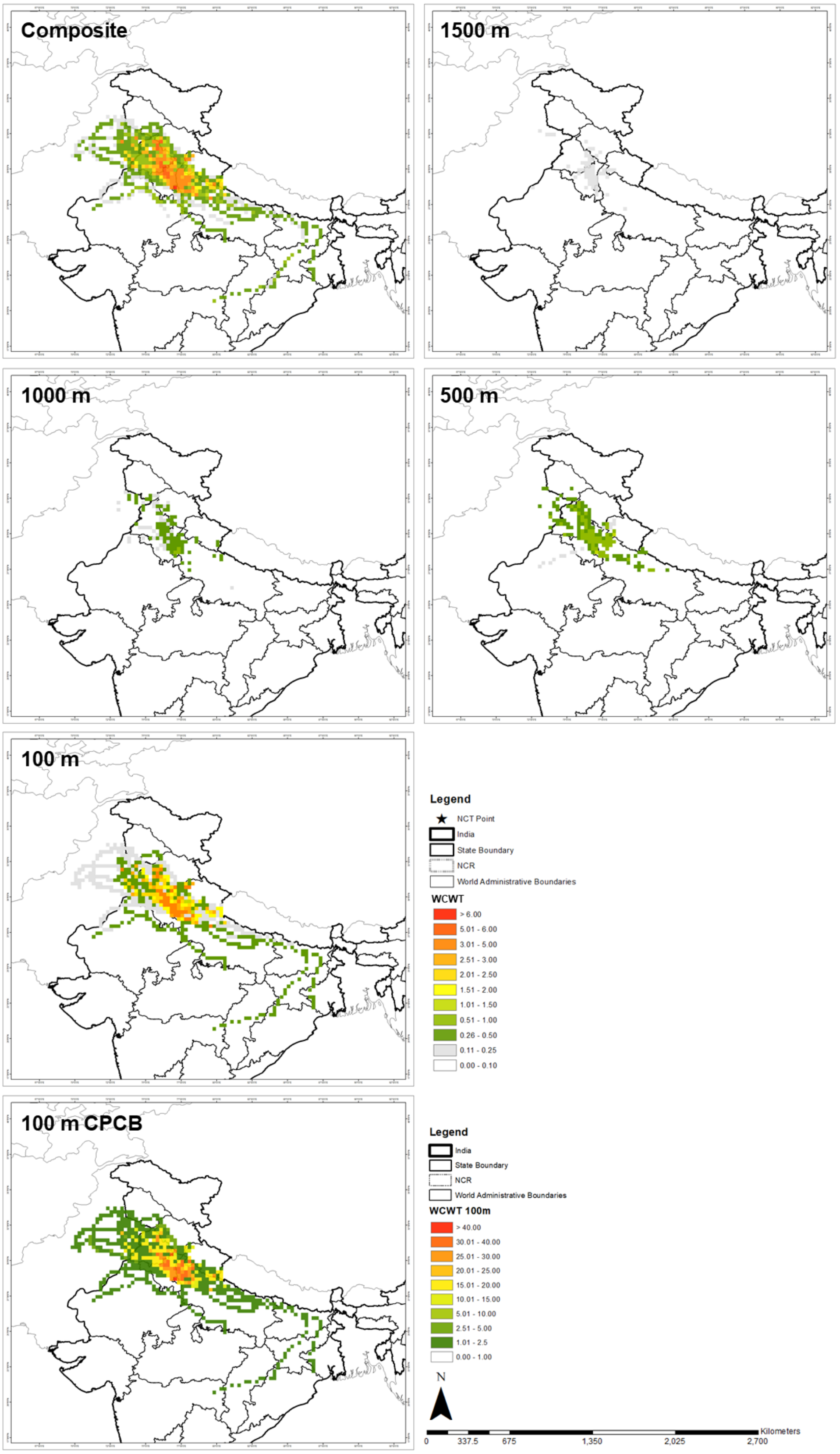

Supplementary Figure S21: PrM WCWT results for NO2 using CAMS and CPCB data at varying heights and composite result

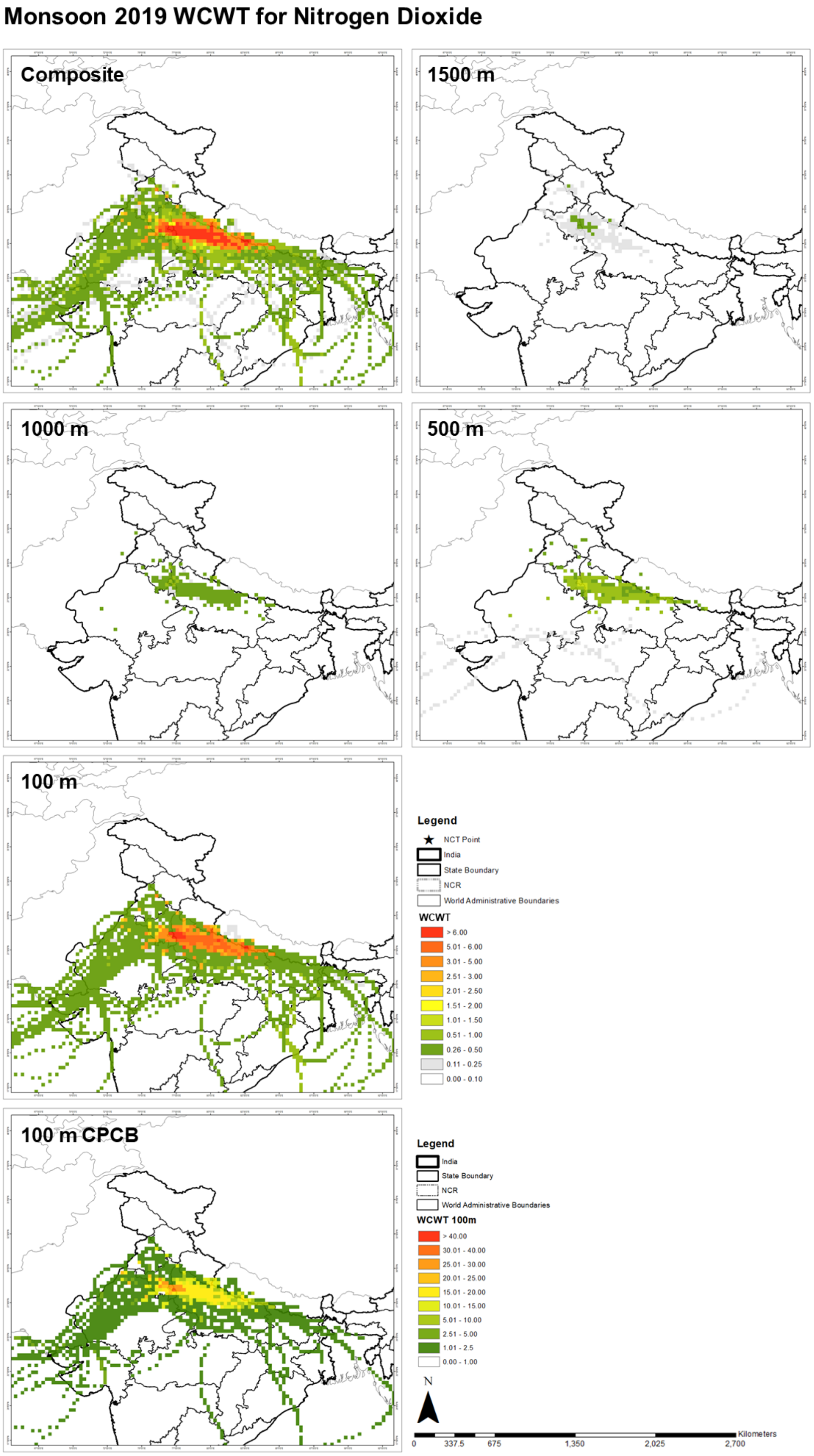

Supplementary Figure S22: Monsoon WCWT results for NO2 using CAMS and CPCB data at varying heights and composite result

Post-Monsoon 2019 WCWT for Nitrogen Dioxide

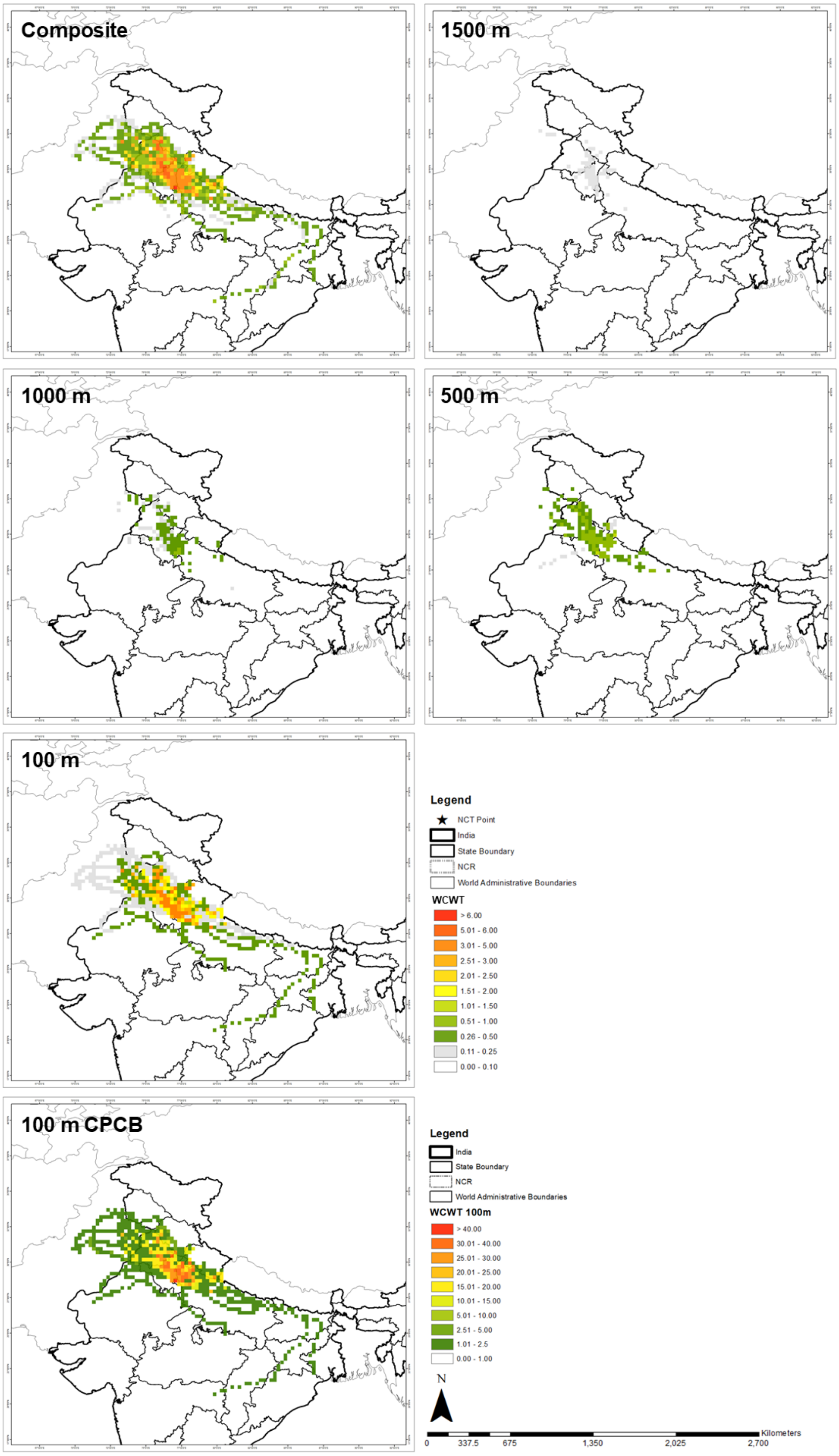

Supplementary Figure S23: Post-Monsoon WCWT results for NO2 using CAMS and CPCB data at varying heights and composite result

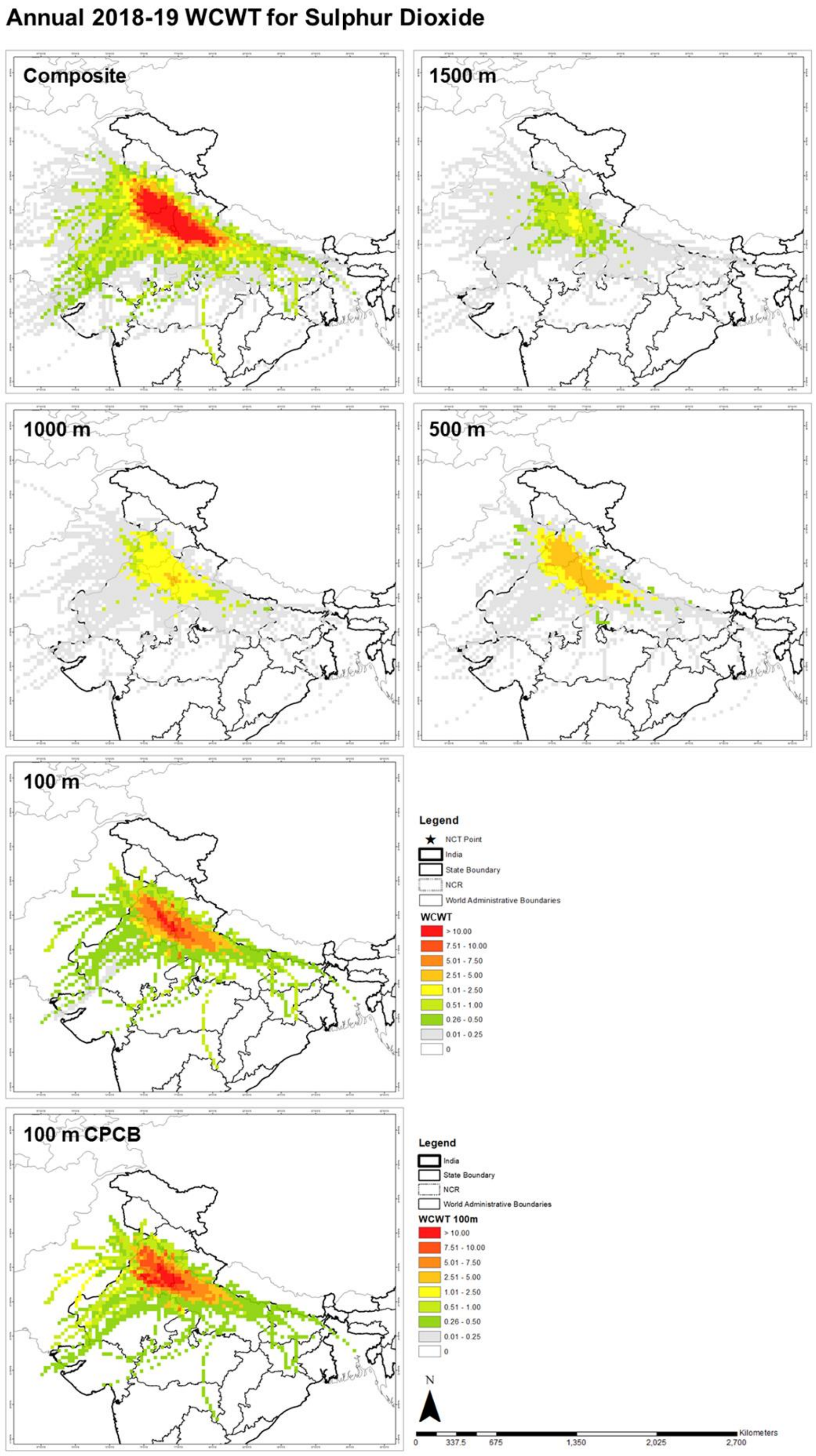

Supplementary Figure S24: Annual WCWT results for SO2 using CAMS and CPCB data at varying heights and composite result

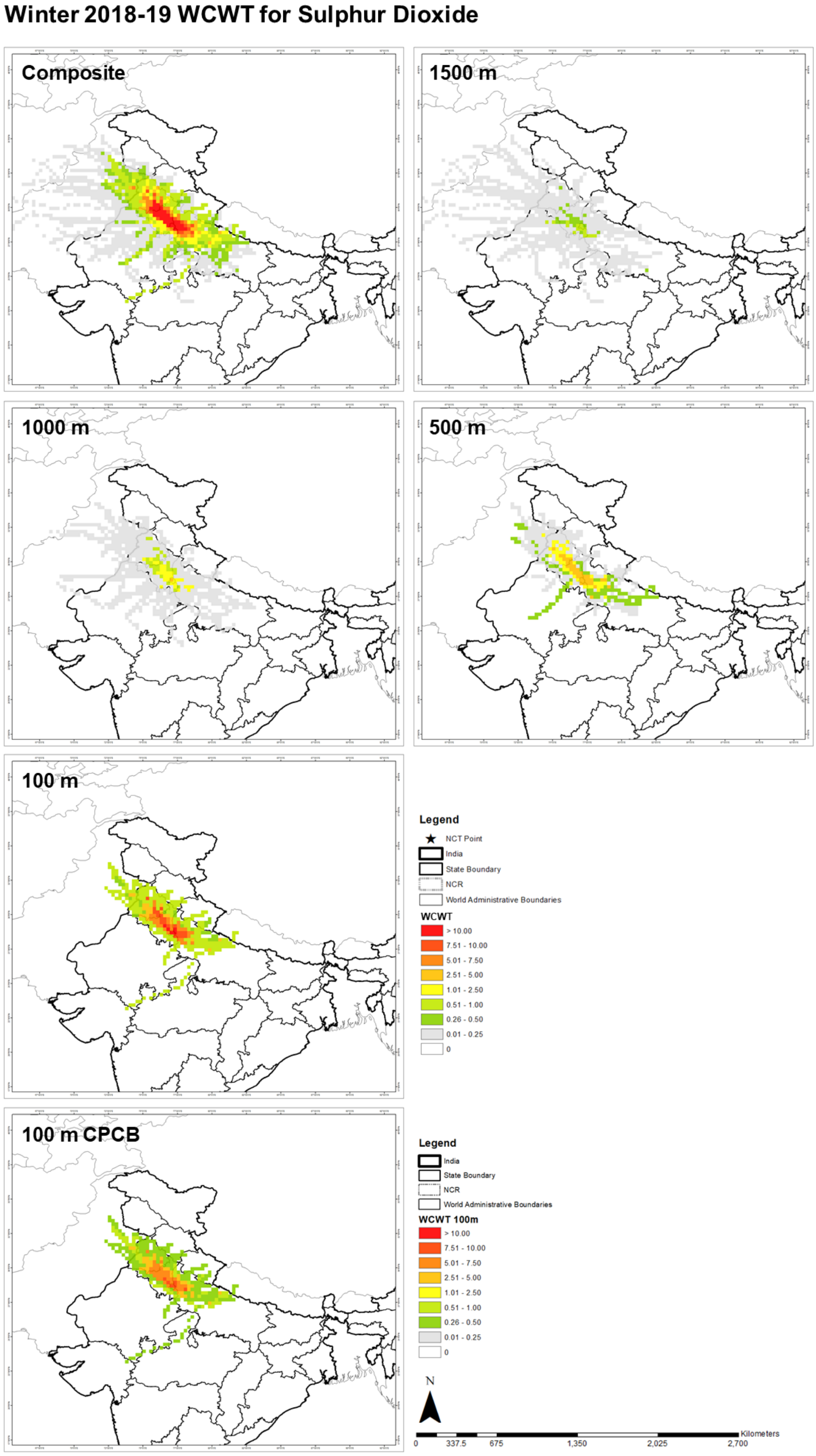

Supplementary Figure S25: Winter WCWT results for SO2 using CAMS and CPCB data at varying heights and composite result

Pre-Monsoon 2019 WCWT for Sulphur Dioxide

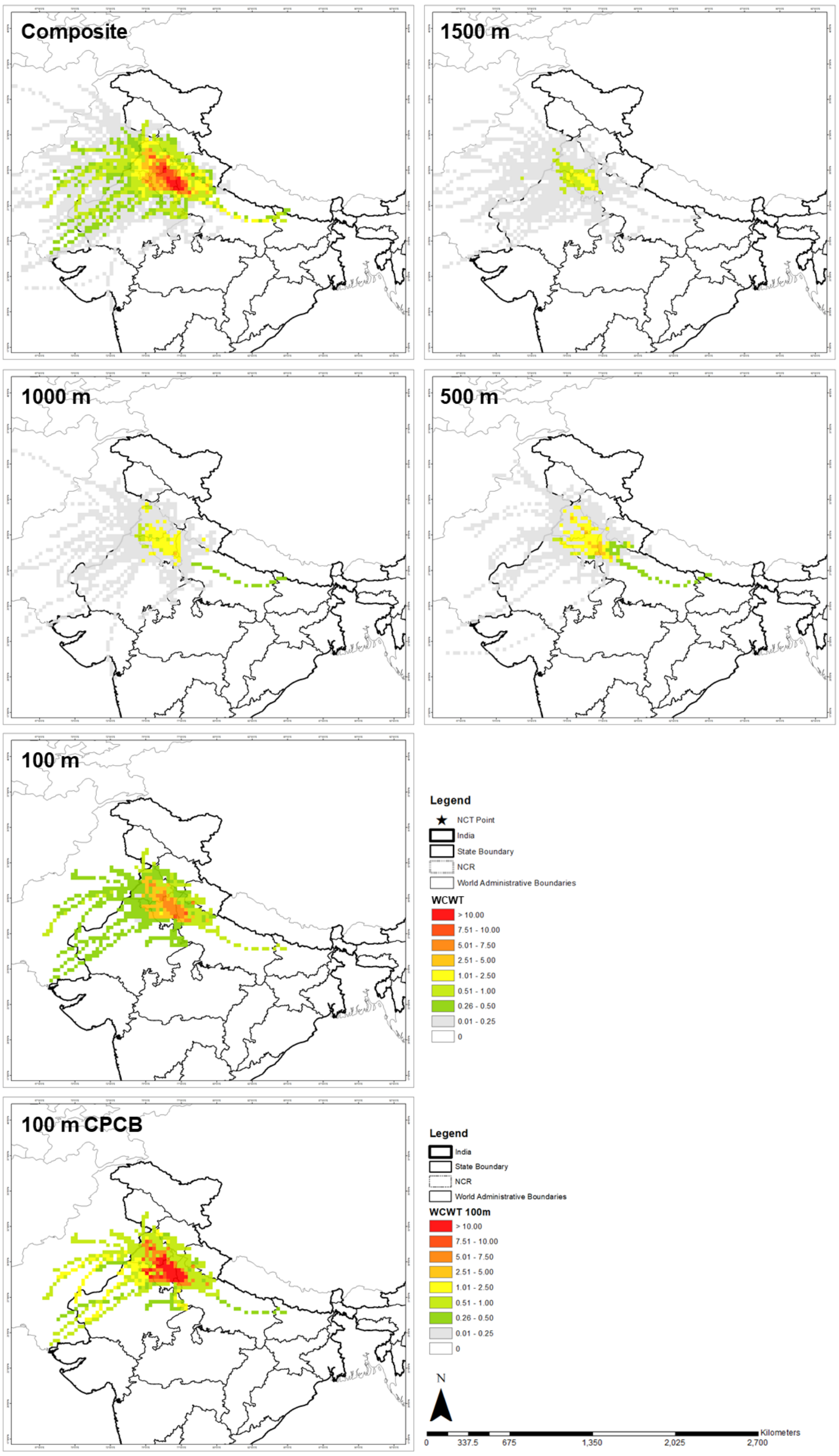

Supplementary Figure S26: PrM WCWT results for SO2 using CAMS and CPCB data at varying heights and composite result

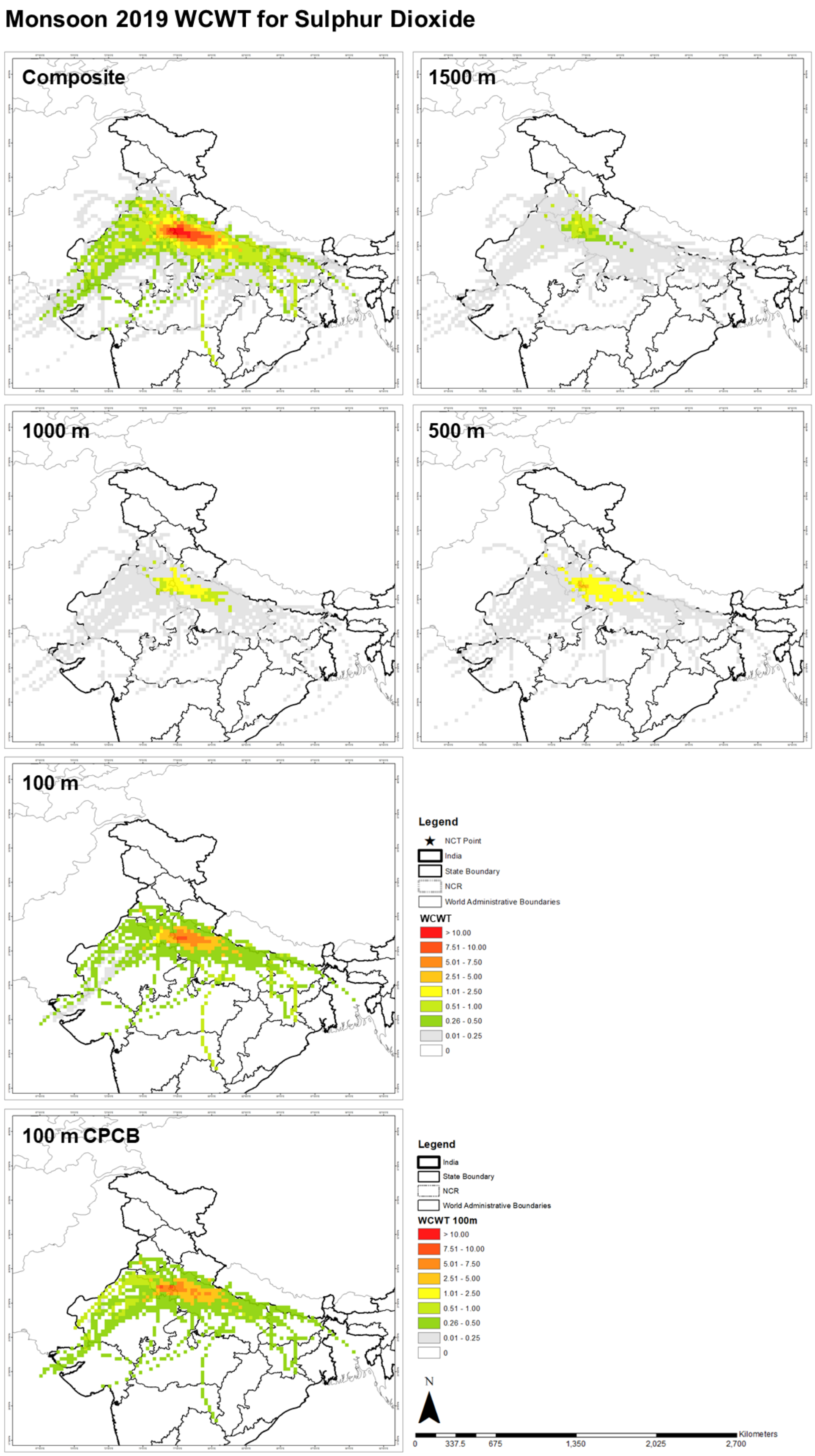

Supplementary Figure S27: Monsoon WCWT results for SO2 using CAMS and CPCB data at varying heights and composite result

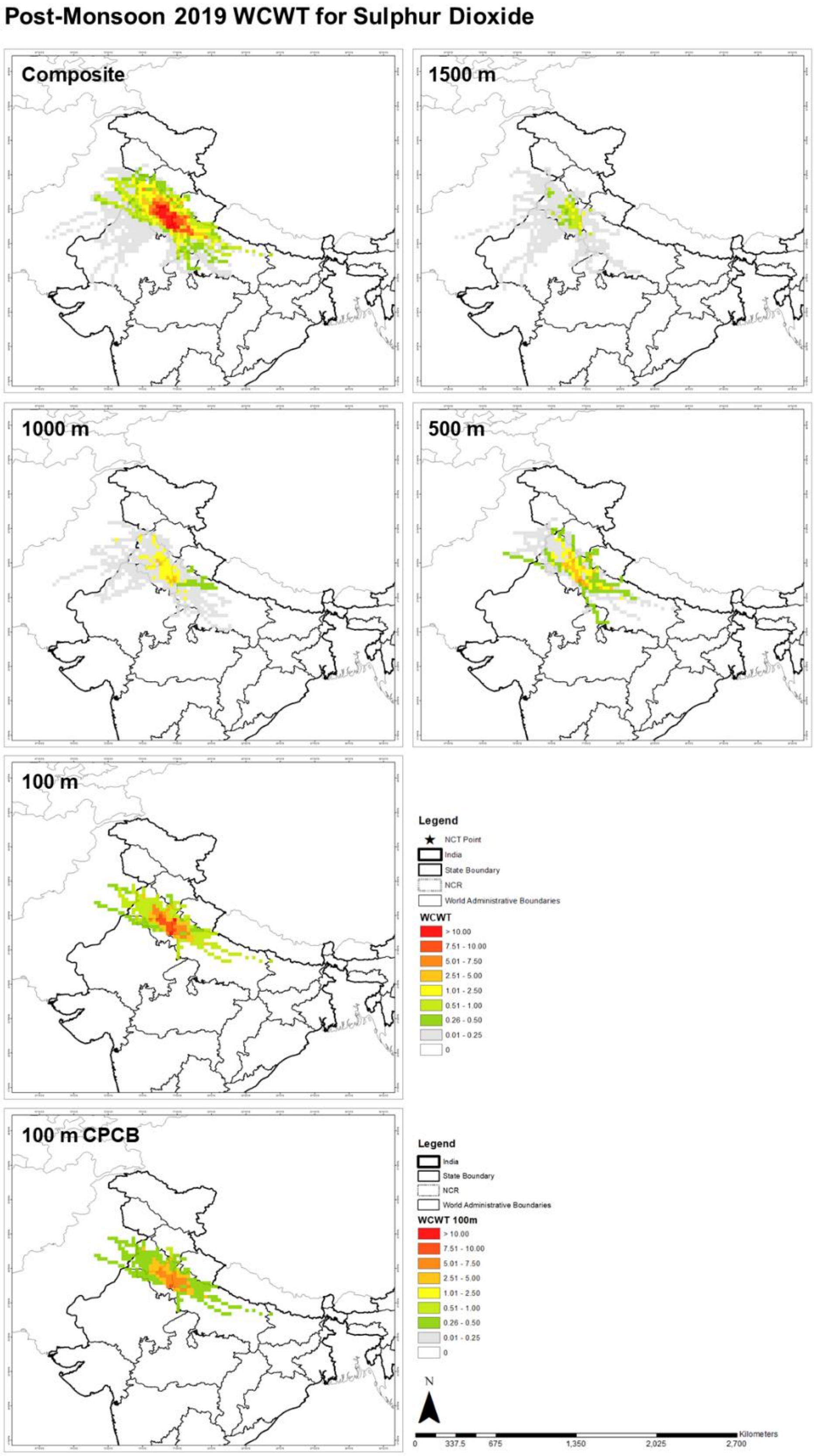

Supplementary Figure S28: PoM WCWT results for SO2 using CAMS and CPCB data at varying heights and composite result

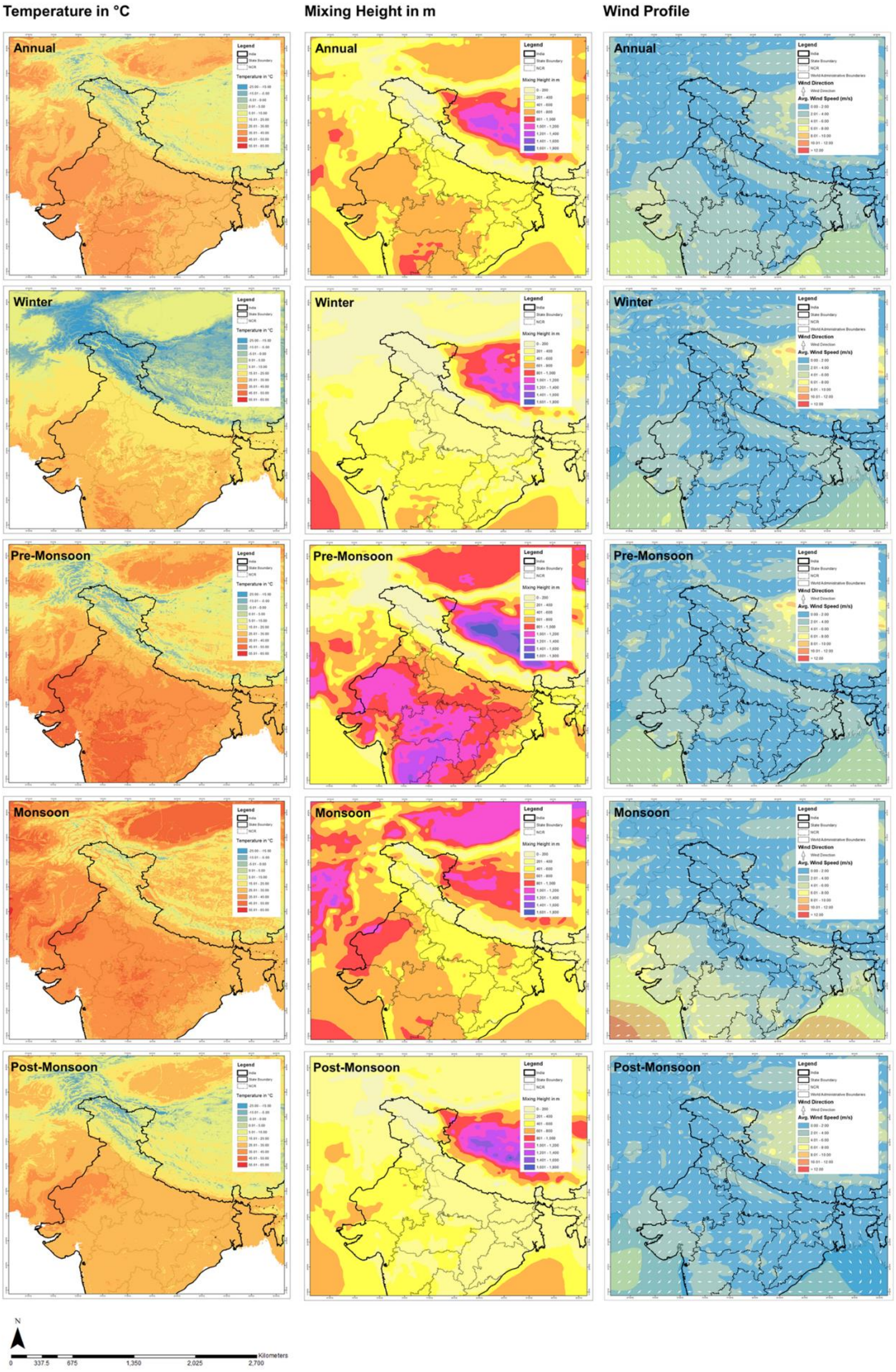

Supplementary Figure S29: Annual and Seasonal average Temperature (MODIS), Mixing Height (ERA 5) and Wind Profile (ERA 5) for 2019
